# riboWaltz: Optimization of ribosome P-site positioning in ribosome profiling data

**DOI:** 10.1371/journal.pcbi.1006169

**Published:** 2018-08-13

**Authors:** Fabio Lauria, Toma Tebaldi, Paola Bernabò, Ewout J. N. Groen, Thomas H. Gillingwater, Gabriella Viero

**Affiliations:** 1 Institute of Biophysics, CNR Unit at Trento, Trento, Italy; 2 Centre for Integrative Biology, University of Trento, Trento, Italy; 3 Euan MacDonald Centre for Motor Neurone Disease Research, University of Edinburgh, Edinburgh, United Kingdom; 4 Centre for Integrative Physiology, University of Edinburgh, Edinburgh, United Kingdom; University of Technology Sydney, AUSTRALIA

## Abstract

Ribosome profiling is a powerful technique used to study translation at the genome-wide level, generating unique information concerning ribosome positions along RNAs. Optimal localization of ribosomes requires the proper identification of the ribosome P-site in each ribosome protected fragment, a crucial step to determine the trinucleotide periodicity of translating ribosomes, and draw correct conclusions concerning where ribosomes are located. To determine the P-site within ribosome footprints at nucleotide resolution, the precise estimation of its offset with respect to the protected fragment is necessary. Here we present riboWaltz, an R package for calculation of optimal P-site offsets, diagnostic analysis and visual inspection of ribosome profiling data. Compared to existing tools, riboWaltz shows improved accuracies for P-site estimation and neat ribosome positioning in multiple case studies. riboWaltz was implemented in R and is available as an R package at https://github.com/LabTranslationalArchitectomics/RiboWaltz.

This is a *PLOS Computational Biology* Software paper.

## Introduction

Ribosome profiling (RiboSeq) is an experimental technique used to investigate translation at single nucleotide resolution and genome-wide scale [1,2], through the identification of short RNA fragments protected by ribosomes from nuclease digestion [3,4]. The last few years have witnessed a rapid adoption of this technique and a consequent explosion in the volume of RiboSeq data [5,6]. In parallel, a number of dedicated computational algorithms were developed for extracting transcript-level information, including unannotated open reading frames (ORFs) [7–10], novel translation initiation sites and differentially translated genes [11,12], as well as positional information describing fluxes of ribosomes along the RNA at sub-codon resolution [13–15] and conformational changes in ribosomes during the elongation step of translation [16].

**Fig 1:**
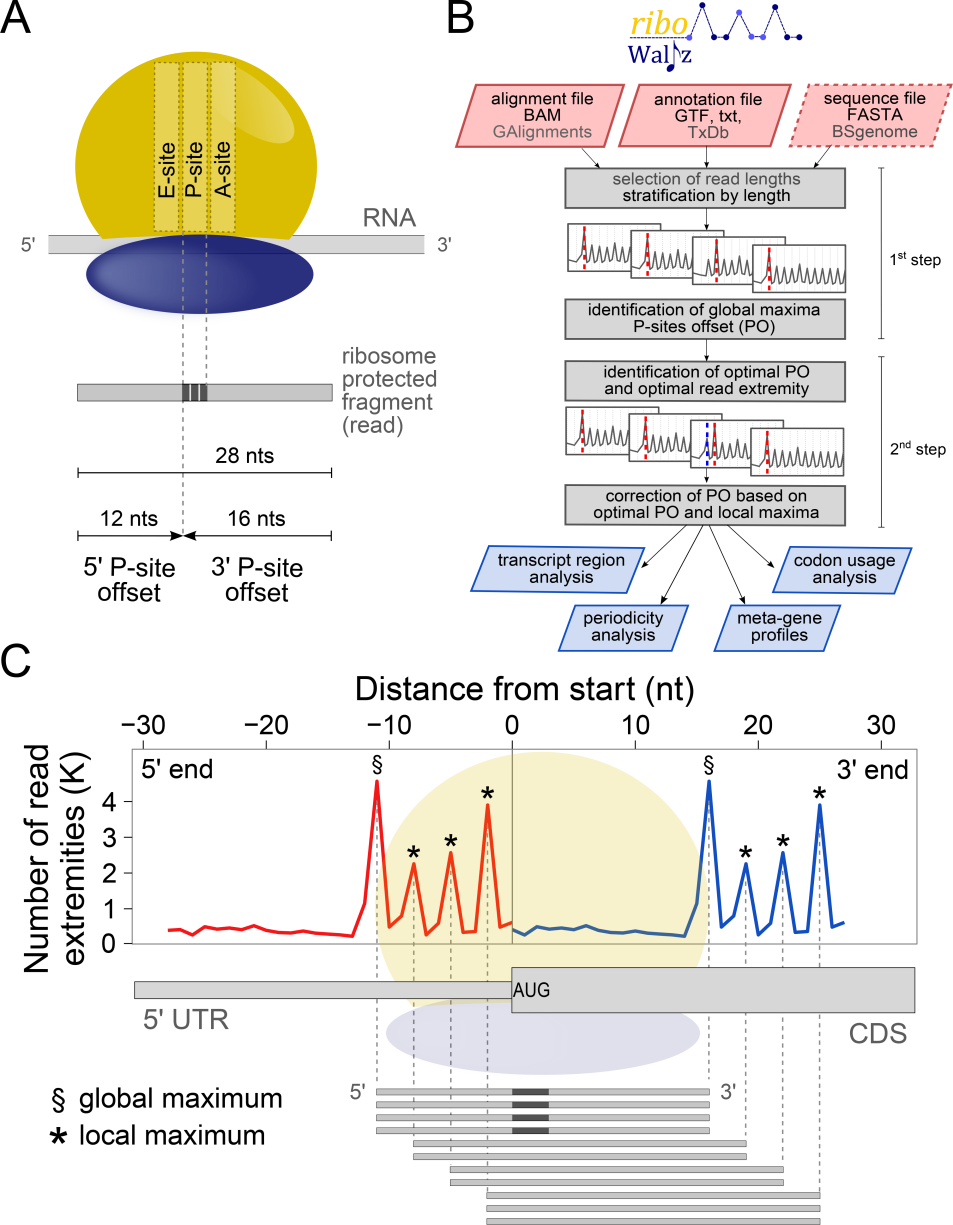
(**A**) Schematic representation of the P-site offset. Two offsets can be defined, one for each extremity of the read. (**B**) Flowchart representing the basic steps of riboWaltz, the input requirements and the outputs. (**C**) An example of ribosome occupancy profile obtained from the alignment of the 5’ and the 3’ end of reads around the start codon (reads length, 28 nucleotides) is superimposed to the schematic representations of a transcript, a ribosome positioned on the translation initiation site (TIS) and a set of reads used for generating the profiles.

Much of this information relies on the ability to determine the exact localization of the P-site, i.e. the site holding the t-RNA associated to the growing polypeptide chain during translation, within ribosome protected fragments (RPF, also called reads hereinafter, following the notation adopted by [1]). This position can be specified by the distance of the P-site from both 5’ and 3’ ends of the reads, the so-called P-site Offset, PO (**Fig 1A**).

Accurate determination of the PO is a crucial step to verify the trinucleotide periodicity of ribosomes along coding regions [1,17], derive reliable translation initiation and elongation rates [18,19], accurately estimate codon usage bias and translation pauses [15,20–23], and reveal novel translated regions in known protein coding transcripts or ncRNAs [8,24,25].

Typically, the PO is defined as a constant number of nucleotides from either the 3' or 5' end of reads, independently from their length (**Fig 1A**) [26]. This approach may lead to an inaccurate detection of the P-site’s position owing to potential offset variations associated with the length of the reads due to different ribosome conformations [16], non-translating ribosomes [27], nuclease digestion biases [15] and sequencing biases [2]. This problem is frequently resolved by selecting subsets of reads with defined length [28,29]. As such, this procedure removes from the analysis reads that are potentially derived from fragments associated to alternative conformations of the ribosome [30,31] and characterized by shorter or longer lengths [16]. Recently, computational tools have been developed to assist with RiboSeq analysis and P-site localization; examples are Plastid [32] and RiboProfiling [33]. Both tools compute the PO after stratifying the reads in bins, according to their length. However, each bin is treated independently, possibly leading to excessive variability of the offsets across bins.

Here, we describe the development of riboWaltz, an R package aimed at computing the PO for all reads from single or multiple RiboSeq samples. Taking advantage of a two-step algorithm, where offset information is passed through populations of reads with different length to maximize the offset coherence, riboWaltz computes with extraordinary precision the PO and shows higher accuracy and specificity of P-site positions than the other methods. riboWaltz provides the user with a variety of graphical representations, laying the foundations for further accurate RiboSeq analyses and better interpretation of positional information.

## Design and implementation

### Input acquisition and processing

riboWaltz is an R package that requires two mandatory input data files: 1) alignment files, in BAM format or as GAlignments objects in R, ideally from transcriptome alignments of RiboSeq reads, and; 2) transcript annotation files, in GTF/GFF3 format or provided as TxDb objects in R. Alternatively, annotation can also be provided as a tab separated text file containing minimal transcript annotation: the length of the transcripts and of their annotated coding sequences and UTRs (**Fig 1B**). Optionally, a third file containing transcript sequence information in FASTA format can be provided as input to perform P-site specific codon sequence analysis. The user is also free to specify a genome build and the corresponding BSGenome object in R will be used for sequence retrieval (**Fig 1B**).

riboWaltz acquires BAM files and converts them into BED files utilizing the *bamtobed* function of the BEDTools suite [34].

### Selection of read lengths

Different lengths of RPFs may derive from alternative ribosome conformations [16,30,31]. Therefore, the researcher should be free to modify the tolerance for the selection of the read length according to the aim of the experiment. For this reason, riboWaltz has multiple options for treating read lengths: i) all read lengths are included in the analysis (all-inclusive mode) ii) only read lengths specified by the user are included (manual mode); iii) only read lengths satisfying a periodicity threshold are included in the analysis (periodicity threshold mode). The user can change the desired threshold (the default is 50%). This mode enables the removal of all the reads without periodicity, similarly to other approaches [10,35].

### Identification of the P-site position

The identification of the P-site, defined by the position of its first nucleotide within the reads, is based on reads aligning across annotated translation initiation sites (TIS or start codon), as proposed by [1]. It is known that the P-site of the reads protected by ribosomes in translation initiation corresponds exactly to the start codon. Thus the P-site offset can be defined as the distance between the extremities of the reads and the start codon itself. After the identification of the P-site for the reads aligning on the TIS, the POs corresponding to each length are assigned to each read of the dataset.

riboWaltz specifically infers the PO in two-steps. First, riboWaltz groups the reads mapping on the TIS according to their length. Each group of reads with a specific length (L) corresponds to a bin. To avoid biases in PO calculation, reads whose extremities are too close to the start codon (9 nucleotides by default) are discarded from the computation of the PO. This parameter, called “flanking length” (FL), can be set by the user. Next, for each length bin, riboWaltz generates the occupancy profiles of read extremities, i.e. the number of 5’ and 3’ read ends in the region around the start codon (**Fig 1C**). For each bin, temporary 5’ and 3’ POs (tPO_L_) are defined as the distances between the first nucleotide of the TIS and the nucleotide corresponding to the global maximum found in the profiles of the 5’ and the 3’ end at the left and at the right of the start codon, respectively (**Fig 1C**). Therefore, considering the occupancy profile as a function *f* of the nucleotide position *x* with respect to the TIS, the temporary 5’ and 3’ POs for each length bin are such that:

$$f({-5\prime tPO}_{L})\geq f(x)\;\forall x\in [-L+FL,-FL]$$

$$f({3\prime tPO}_{L})\geq f(x)\;\forall x\in [FL-1,L-FL-1]$$

The two sets of length-specific temporary POs are defined as:

$$5\prime tPO=\{{5^{\prime }tPO}_{L_{min}},\ldots ,{5^{\prime }tPO}_{L_{max}}\}$$

$$3\prime tPO=\{{3^{\prime }tPO}_{L_{min}},\ldots ,{3^{\prime }tPO}_{L_{max}}\}$$

where *L*_*min*_ and *L*_*max*_ are the minimum and the maximum length of the reads, respectively.

Next, to each read (R) mapping on the TIS the temporary POs corresponding to its length is assigned, obtaining two sets of read-specific tPOs:

$${5^{\prime }tPO}_{R}=\{{5^{\prime }tPO}_{R_{1}},\ldots ,{5^{\prime }tPO}_{R_{N}}\}$$

$${3^{\prime }tPO}_{R}=\{{3^{\prime }tPO}_{R_{1}},\ldots ,{3^{\prime }tPO}_{R_{N}}\}$$

where N is the number of reads mapping on the TIS.

Despite good estimation of P-site positions, artifacts may arise from either the small number of reads with a specific length or the presence of reads from ribosomes nearby the TIS, but not translating the first codon. In other words, the offset estimated independently from the global maximum of each read length is not necessarily always the best choice. In fact, while the most abundant population of reads are less subjected to the above mentioned biases and show consistent tPOs (see **S1–S12 Text**), this approach can produce high variability in tPO_L_ values of reads differing in only one nucleotide in length, especially across length bins with low number of reads.

To minimize this problem, riboWaltz exploits the most frequent tPO (optimal PO: oPO) associated to the predominant bins as a reference value for correcting the temporary POs of smaller bins. Briefly, the correction step defines for each length bin a new PO based on the local maximum, whose distance from the TIS is the closest to the oPO. The complete procedure is illustrated below.

The optimal PO at either 5’ or 3’ extremities (optimal extremity) are chosen as reference points to adjust the other tPOs. The optimal PO is selected between the two modes of read specific tPO sets (*Mode*(5′*tPO*_*R*_) and *Mode*(3′*tPO*_*R*_)) as the one with the highest frequency.

$$oPO≔\left\{ \begin{array}{l} Mode({5^{\prime }tPO}_{R})\;if\;frequency(Mode({5^{\prime }tPO}_{R}))\geq frequency(Mode({3^{\prime }tPO}_{R}))\\ Mode({3^{\prime }tPO}_{R})\;if\;frequency(Mode({5^{\prime }tPO}_{R}))<frequency(Mode({3^{\prime }tPO}_{R})) \end{array}\right.$$

Note that this step also selects the optimal extremity to calculate the corrected PO.

The correction step is specific for each bin length and works as follows: if the offset associated to a bin is equal to the optimal PO, no changes are made. Otherwise, i) the local maxima of the occupancy profiles are extracted; ii) the distances between the first nucleotide of the TIS and each local maxima is computed; iii) the corrected PO is defined as the distance in point ii) that is closest to the optimal PO. Summarizing, given the set of local maxima positions (LMP) of the occupancy profile for the optimal extremity, the corrected PO for reads of length L (*cPO*_*L*_) satisfies the following condition:

$${cPO}_{L}-oPO=\underset{x\in \mathrm{LMP}}{\min}\left(x-oPO\right)$$

### Output

riboWaltz returns three data structures that can be used for multiple downstream analysis workflows (**Fig 1B**). The first is a list of sample-specific data frames containing for each read i) the position of the P-site (identified by the first nucleotide of the codon) with respect to the beginning of the transcript; ii) the distance between the P-site and both the start and the stop codon of the coding sequence; iii) the region of the transcript (5' UTR, CDS, 3' UTR) where the P-site is located and iv) the sequence of the triplet covered by the P-site, if a sequence file is provided as input. The second data structure is a data frame with the percentage of reads aligning across the start codon (if any) and along the whole transcriptome, stratified by sample and read length. Moreover, this file includes the P-site offsets from both the 5’ and 3’ extremities before and after the optimization (5' tPO_L_, 3' tPO_L_, 5' cPO_L_, 3' cPO_L_ values). The third data structure is a data frame containing, for each transcript, the number of estimated in-frame P-sites on the CDS. This data frame can be used to estimate transcript-specific translation levels and to perform differential analysis comparing multiple samples in different conditions.

In addition, riboWaltz provides several graphical outputs based on the widely used “ggplot2” package. riboWaltz plots are described in more detail in the Results section. All graphical outputs are returned as lists containing objects of class “ggplot”, further customizable by the user, and data frames containing the source data for the plots.

## Results

### riboWaltz overview

To illustrate the functionalities of riboWaltz, we analyzed seven ribosome profiling datasets in yeast, mouse and human samples (see **Figs 2 and 3** and **S1–S13 Figs**).

**Fig 2:**
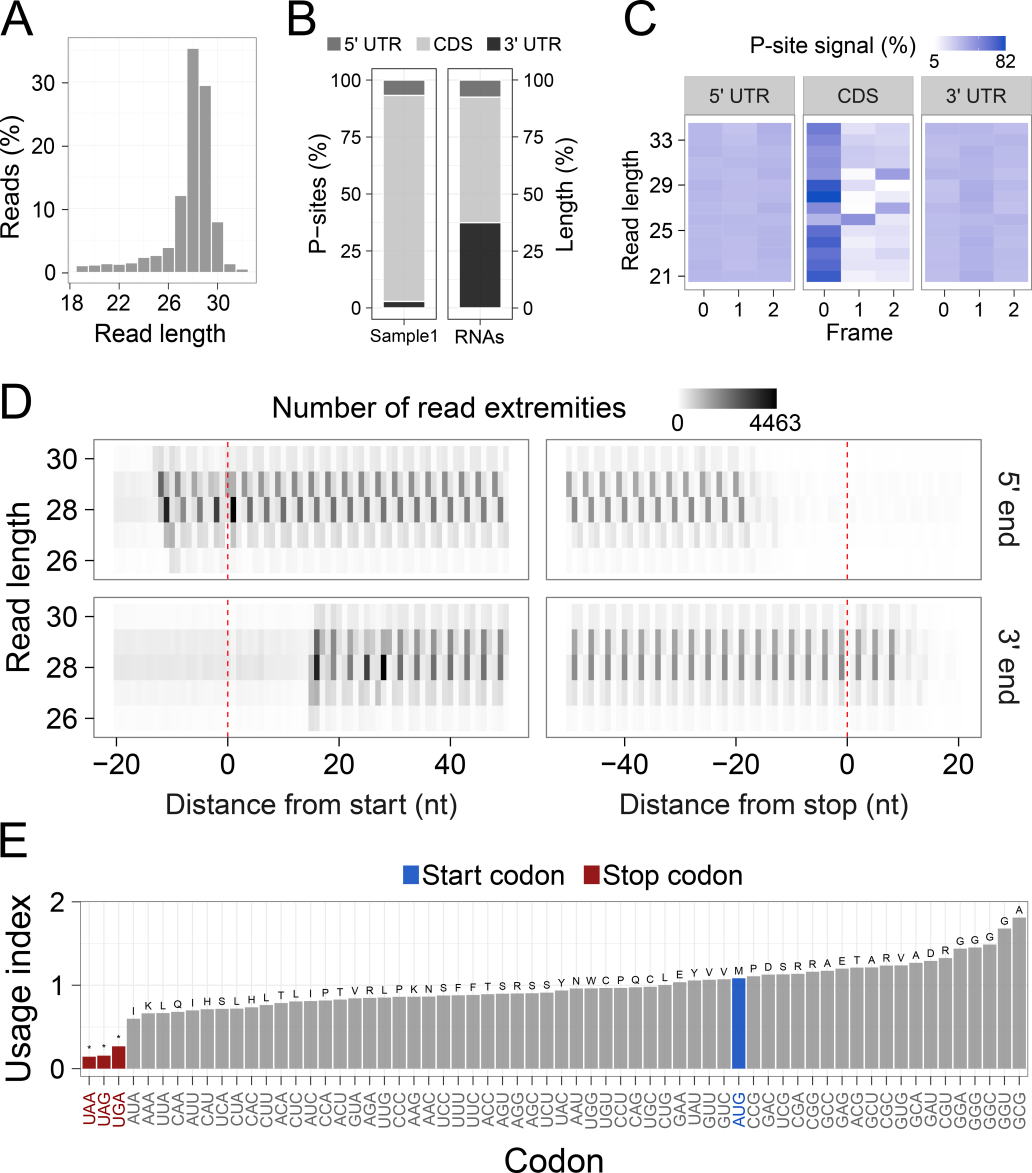
(**A**) Distribution of the read lengths. (**B**) Left, percentage of P-sites in the 5’ UTR, CDS and 3’ UTR of mRNAs from ribosome profiling data. Right, percentage of region lengths in mRNAs sequences. (**C**) Percentage of P-sites in the three frames along the 5’ UTR, CDS and 3’ UTR, stratified for read length. (**D**) Example of meta-gene heatmap reporting the signal associated to the 5’ end (upper panel) and 3’ end (lower panel) of the reads aligning around the start and the stop codon for different read lengths. (**E**) Codon usage analysis based on in-frame P-sites. The codon usage index is calculated as the frequency of in-frame P-sites along the coding sequence associated to each codon, normalized for codon frequency in sequences. The amino-acids corresponding to the codons are displayed above each bar. All panels were obtained from ribosome profiling of whole mouse brain (GSE102318).

**Fig 3:**
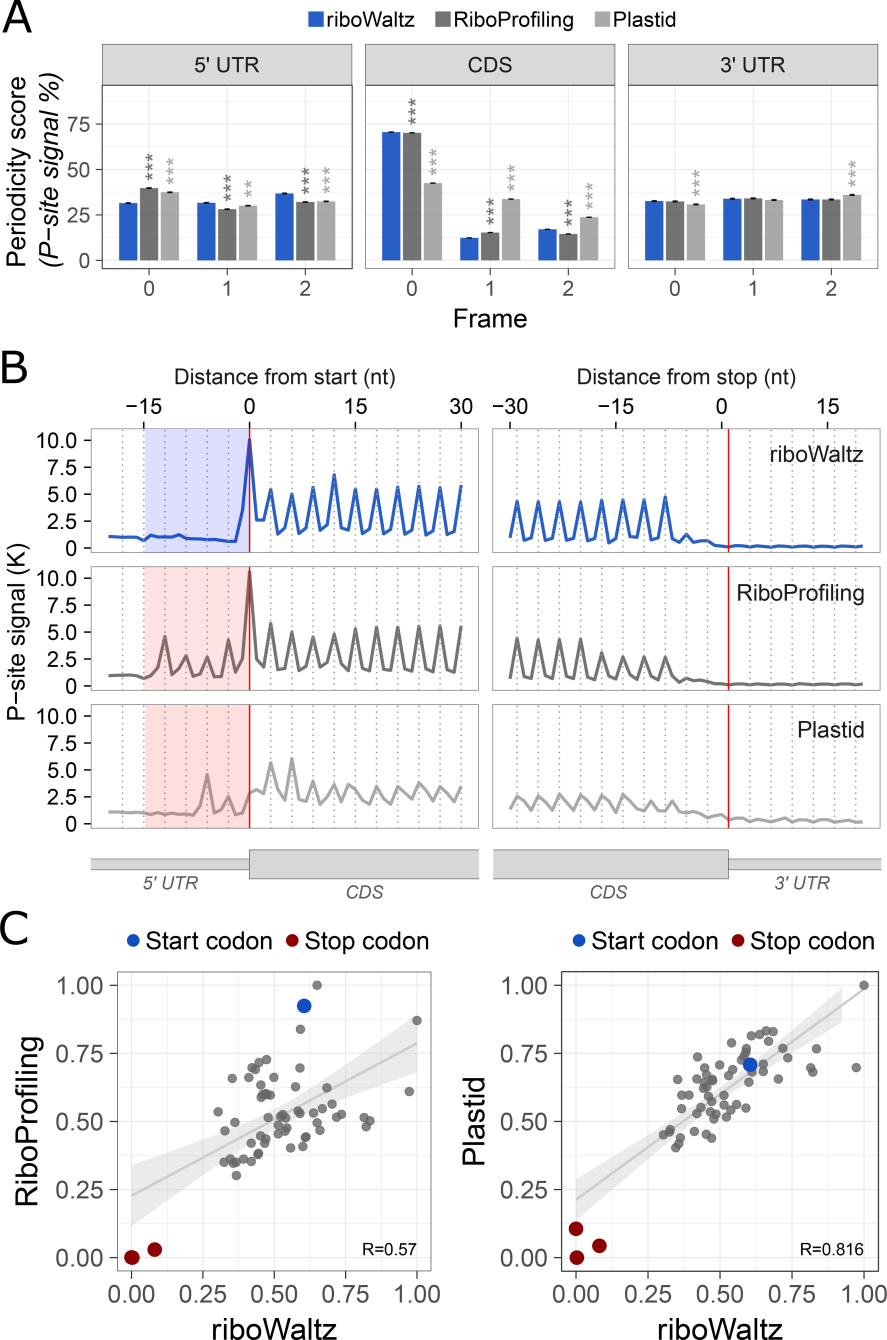
(**A**) Percentage of P-sites in the three frames (Periodicity score) along the 5’ UTR, CDS and 3’ UTR from ribosome profiling performed in mouse brain (GSE102318). The statistical significances from two-tailed Wilcoxon–Mann–Whitney test comparing RiboProfiling and Plastid with respect to riboWaltz are reported (P-value: ** < 0.01, *** < 0.001). (**B**) Meta-profiles showing the periodicity of ribosomes along the transcripts at the genome-wide scale. The three metaprofiles are based on the P-site identification obtained by using riboWaltz, RiboProfiling and Plastid. The shaded areas to the left of the start codon highlight the shift of the periodicity toward the 5’ UTR that is absent in the case of data analysed using riboWaltz. (**C**) Comparison between the codon usage index based on in-frame P-sites from riboWaltz and RiboProfiling (left panel) and between the codon usage index based on in-frame P-sites from riboWaltz and Plastid (right panel). The length of the reads ranges from 19 up to 38 nucleotides (see Table 1) with the optimal PO used in the correction step of riboWaltz being 16 nucleotides from the 3’ end.

riboWaltz integrates several graphical functions that provide multiple types of output results. First, the distribution of the length of the reads (**Fig 2A**): this is a useful preliminary inspection tool to understand the contribution of each bin to the final P-site determination, and eventually decide to remove certain bin from further analyses. Second, the percentage of P-sites located in the 5’ UTR, CDS and 3’ UTR regions of mRNAs compared to a uniform distribution weighted on region lengths, which simulates random P-site positioning along mRNAs (**Fig 2B**). This analysis is a good way to verify the expected enrichment of ribosome signal in the CDS. Third, to understand to which extent the obtained P-sites result in codon periodicity in the CDS, riboWaltz produces for every read group a plot with the percentage of P-sites in the three possible translation reading frames (periodicity analysis) for 5’ UTR, CDS and 3’ UTR (**Fig 2C**). Fourth, riboWaltz returns for every read length the meta-gene read density heatmap for both the 5’ and 3’ extremities of the reads (**Fig 2D**). This plot provides an overview of the occupancy profiles used for P-site determination and allows the visual inspection of PO values reliability. Fifth, to understand what codons display higher or lower ribosome density, riboWaltz provides the user with the analysis of the empirical codon usage, i.e. the frequency of in-frame P-sites along the coding sequence codon by codon, normalized for the frequency of each codon in the sequences (**Fig 2E**). Indeed, the comparison of these values in different biological conditions can be of great help to unravel possible defects in ribosome elongation at specific codons or aa-tRNAs use. Finally, single transcripts profiles and meta-gene profiles based on P-site position can be generated (**Fig 3B, top row** see **S1–S13 Figs** for examples) with multiple options: i) combining multiple replicates applying convenient scale factors provided by the user, ii) considering each replicate separately, or iii) selecting a subsets of reads with defined length.

### Comparison with other tools

**Fig 4:**
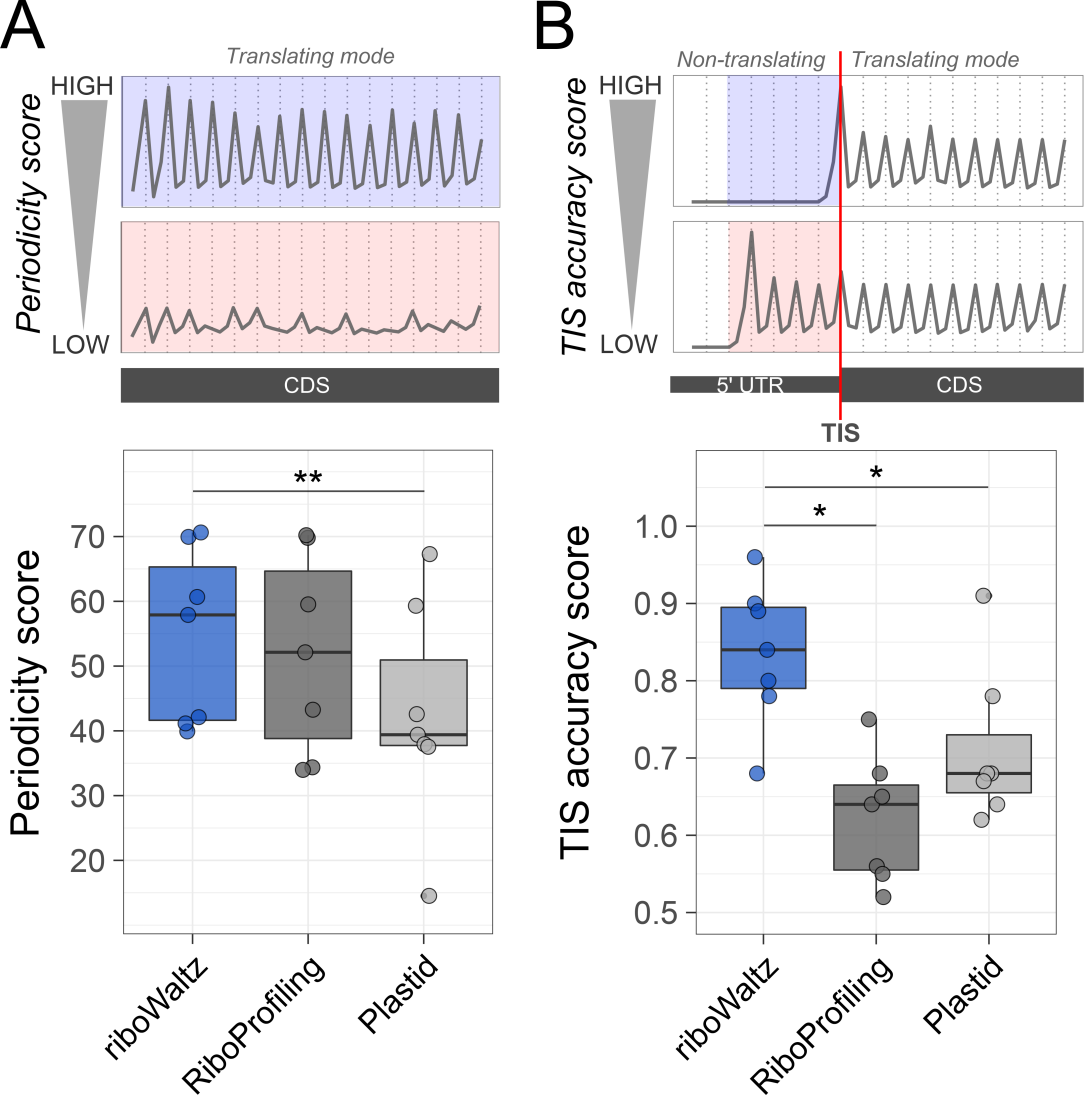
(**A**) Comparison of the percentage of P-sites in frame 0 (Periodicity score) along the coding sequence and (**B**) comparison of the average TIS accuracy score based on P-sites identification by riboWaltz, RiboProfiling and Plastid. Both panels display the results obtained from 7 datasets (2 yeast, 3 mouse and 2 human), each dataset represented by a dot. Statistical significances from paired one-tailed Wilcoxon–Mann–Whitney test are shown (* P<0.05, ** P<0.01).

We tested riboWaltz on multiple ribosome profiling datasets in different model organisms: yeast (*S*. *cerevisiae*, [16,36]), mouse (mESC, [37]; whole brain, GSE102318) and human samples (Hek-293 [26]; MCF-7, GSE111866) and compared riboWaltz, RiboProfiling (v1.2.2, [33]) and Plastid (v0.4.5, [32]). Both Plastid and RiboProfiling compute the P-site offset considering the highest peak in the profile of reads mapping around the translation initiation site (TIS). Differently from RiboProfiling, Plastid considers only the signal from the 5’ end of the read and imposes a default threshold for the minimum number of reads required for the computation. If this requirement is not met, Plastid will use a "default" constant offset value. **Table 1** and **S1–S6 Texts** contain the P-site offset comparison between the three tools, while **Table 2** and **S7–S12 Texts** provide additional details on the offsets computed by riboWaltz. The three tools were run using default settings. The comparisons for single datasets are displayed in **Fig 3** and in **S1–S6 Figs**, while the summary and the evaluation of the comparisons for all the datasets are displayed in **Fig 4**.

**Table 1:** Comparison of the P-site offsets identified for each read length by riboWaltz, RiboProfiling and Plastid in mouse (GSE102318).

Read length	riboWaltz		RiboProfiling		Plastid	
	from 5’ end	from 3’ end	from 5’ end	from 3’ end	from 5’ end	from 3’ end
**19**	2	16	2	16	13	5
**20**	4	15	4	15	13	6
**21**	4	16	4	16	13	7
**22**	5	16	5	16	13	8
**23**	6	16	6	16	13	9
**24**	7	16	7	16	13	10
**25**	8	16	1	25	13	11
**26**	10	15	10	15	13	12
**27**	10	16	10	16	13	13
**28**	11	16	1	28	5	22
**29**	12	16	12	16	13	15
**30**	12	17	10	19	35	6
**31**	13	17	20	50	13	17
**32**	15	16	15	16	13	18
**33**	16	16	17	15	13	19
**34**	17	16	17	16	13	20
**35**	18	16	18	16	13	21
**36**	16	19	19	16	13	22
**37**	20	16	22	58	13	23
**38**	21	16	15	22	13	24

The POs computed from both read extremities are reported. The optimal PO used in the correction step of riboWaltz corresponds to 16 nucleotides from the 3’ end.

**Table 2:** Comparison between temporary and corrected P-site offsets identified by riboWaltz in mouse (GSE102318).

**Read** **length**	**Number of reads (%)**	**Temporary P-site offset**		**Corrected P-site offset**	
		from 5’	from 3’	from 5’	from 3’
**19**	0.888	2	16	2	16
**20**	0.986	4	15	4	15
**21**	1.203	4	16	4	16
**22**	1.113	5	16	5	16
**23**	1.335	6	16	6	16
**24**	2.191	7	16	7	16
**25**	2.494	8	16	8	16
**26**	3.743	10	15	10	15
**27**	11.891	10	16	10	16
**28**	34.943	11	16	11	16
**29**	29.125	12	16	12	16
**30**	7.771	12	17	12	17
**31**	1.194	11	19	13	17
**32**	0.365	15	16	15	16
**33**	0.235	16	16	16	16
**34**	0.164	17	16	17	16
**35**	0.115	18	16	18	16
**36**	0.087	10	25	16	19
**37**	0.057	20	16	20	16
**38**	0.034	21	16	21	16

The POs computed from both read extremities are reported. The optimal PO used in the correction step correspond to 16 nucleotides from the 3’ end.

To evaluate the three methods, we considered two performance scores. First, we estimated the percentage of P-sites with correct frame within the CDS region (Periodicity score). The higher this measure, the better the performance. For RiboWaltz and RiboProfiling, this measure was comparable in almost all datasets, while Plastid performed worse (see **Fig 3A** and **S1–S6A Figs** for individual examples, **Fig 4A** and **Table 3** for a resume. The median values are: riboWaltz: 57.07; RiboProfiling: 51.45; Plastid: 39.04).

**Table 3:** Summary and comparison of the percentage of P-sites in frame 0 along the coding sequence (Periodicity score) based on P-sites identification by riboWaltz, RiboProfiling and Plastid.

Organism	Reference	Mean % of P-site in frame 0			Statistical significance	
		riboWaltz	Ribo Profiling	Plastid	riboWaltz vs RiboProfiling	riboWaltz vs Plastid
**Yeast**	Lareau et al., 2014 [16]	42.11	43.26	39.40	5.90·10^−4^ ***	8.99·10^−21^ ***
**Yeast**	Beaupere et al., 2017 [36]	69.95	69.80	67.29	0.0046 **	5.40·10^−124^ ***
**Mouse**	This publication (GSE102318)	70.63	70.21	42.58	1.12·10^−7^ ***	< 1·10^−324^ ***
**Mouse** (IP RPL10)	Shi et al., 2017 [37]	39.91	34.37	37.94	< 1·10^−324^ ***	2.15·10^−125^ ***
**Mouse** (IP RPL22)	Shi et al., 2017 [37]	41.15	33.97	37.54	< 1·10^−324^ ***	4.39·10^−277^ ***
**Human**	Gao et al., 2015 [26]	60.67	59.53	59.31	2.37·10^−15^ ***	1.27·10^−15^ ***
**Human**	This publication (GSE111866)	57.90	52.13	14.52	5.89·10^−191^ ***	< 1·10^−324^ ***

The values obtained from 7 datasets (2 yeast, 3 mouse and 2 human) are shown, together with the statistical significances from two-tailed Wilcoxon–Mann–Whitney test (P-value:

* < 0.05

** < 0.01

*** < 0.001).

Next, we took into consideration the meta-profiles. In all datasets riboWaltz displayed a neat periodicity uniquely in the CDS (**Fig 3B** and **S1–S6B Figs**), with almost no signal along the UTRs, neither in the proximity of the start nor of the stop codons. By contrast, both Plastid and RiboProfiling generated a shift toward the 5’ UTR in the beginning of the periodic region (**Fig 3B** and **S1–S6B Figs**). The presence of periodic peaks in the 5’UTR is undoubtedly a source of biological inaccuracy, conflicting with basic concepts in translation. In fact, outside the coding sequence, ribosomes are generally in non-translating mode. Translation can indeed occur outside the CDS, with upstream ORFs being the most documented examples. Nonetheless, occasional translation outside the CDS is unlikely to affect the codon periodicity in 5’ UTR regions, especially when metagene plots are anchored on the annotated AUG start codons. The presence of prominent codon periodicity in the 5’UTR in this latter case most likely results from a technical mistake, such as the inaccurate computation of the P-site offset. To quantify this effect, we determined a “TIS accuracy score”, comparing the amount of periodic signal in a local window before and after the translation initiation site. Considering the occupancy profile as a function *f* of the nucleotide position *x* with respect to the TIS, the TIS accuracy score is defined as follows:

$$TIS\;accuracy\;score≔\frac{\sum_{\left\{x\in [0,14]:3|x\right\}}f(x)}{\sum_{\left\{x\in [-15,14]:3|x\right\}}f(x)}$$

In the ideal scenario, this score should be equal to 1, meaning that the periodicity can be detected only within the CDS region. Lower scores are associated with a progressive increase of periodicity in the 5’UTR, indicative of ribosome mislocalization. Importantly, riboWaltz shows significantly higher TIS accuracy scores with respect to both RiboProfiling and Plastid (median values: 0.84, 0.62, 0.71 respectively. See **Fig 4B** and **Table 4** for a resume).

**Table 4:** Summary and comparison of the average TIS accuracy score based on P-sites identification by riboWaltz, RiboProfiling and Plastid.

Organism	Reference	Average TIS accuracy score			Statistical significance	
		riboWaltz	Ribo Profiling	Plastid	riboWaltz vs RiboProfiling	riboWaltz vs Plastid
**Yeast**	Lareau et al., 2014 [16]	0.90	0.75	0.91	6.0 ·10^−45^ ***	0.6817
**Yeast**	Beaupere et al., 2017 [36]	0.96	0.56	0.68	< 1·10^−324^ ***	< 1·10^−324^ ***
**Mouse**	This publication (GSE102318)	0.89	0.65	0.68	< 1·10^−324^ ***	< 1·10^−324^ ***
**Mouse** (IP RPL10)	Shi et al., 2017 [37]	0.68	0.56	0.67	1.5 ·10^−98^ ***	0.9015
**Mouse** (IP RPL22)	Shi et al., 2017 [37]	0.78	0.52	0.79	< 1·10^−324^ ***	0.0013 **
**Human**	Gao et al., 2015 [26]	0.84	0.68	0.62	3.4 ·10^−221^ ***	< 1·10^−324^ ***
**Human**	This publication (GSE111866)	0.80	0.65	0.64	3.2 ·10^−78^ ***	1.1 ·10^−50^ ***

The values obtained from 7 datasets (2 yeast, 3 mouse and 2 human) are shown, together with the statistical significances from two-tailed Wilcoxon–Mann–Whitney test (P-value:

* < 0.05

** < 0.01

*** < 0.001).

The correct localization of ribosomes is a crucial step for obtaining estimations of the codon usage and for any downstream analyses. Empirical codon usage determination is a popular analysis for ribosome profiling data, and it is equally important for the biological interpretation of results and for the development of reliable mathematical models of translation [20–22,38–40]. To highlight the differences arising in codon usage after the identification of the P-site using different approaches, we compared codon usage values across each dataset analysed using riboWaltz, RiboProfiling and Plastid (**Fig 3C** and **S1–S6C Figs**). The results show correlation values ranging from 0.075 to 0.999. This analysis is a descriptive evaluation of the difference between riboWaltz and the other tools in computing the codon usage, depending on the different approach used for the P-site determination.

In summary we show that the choice of the strategy for P-site positioning has a strong impact on downstream analyses and that riboWaltz is a more reliable tool for the identification of P-site offsets and the positional analysis of ribosome profiling data.

## Availability and future directions

riboWaltz identifies with high precision the position of ribosome P-sites from ribosome profiling data. By improving on other currently-available approaches, riboWaltz can assist with the detailed interrogation of ribosome profiling data, providing precise information that may lay the groundwork for further positional analyses and new biological discoveries.

riboWaltz is written in the R programming language, and is compatible with Linux, Mac, or Windows PCs. riboWaltz depends on multiple R packages such as GenomicFeatures for handling GTF/GFF3 files, Biostrings, BSgenome and GenomicAlignments for dealing with sequence data and ggplot2 for data visualization. Furthermore, to easily handle datasets with several millions of reads preserving a high efficiency in terms of RAM usage and running-time, riboWaltz employs an enhanced version of data frames provided by the data.table package. Installation instructions for the dependencies are provided in the manual.

riboWaltz is an Open-Source software package that can be extended in future releases to include other analysis methods as they are developed. Source code for riboWaltz is distributed under the MIT license and is available at the following GitHub repository: https://github.com/LabTranslationalArchitectomics/riboWaltz. The package includes the R implementation of riboWaltz, data used in this article, extensive documentation and a stable release.

## Supporting information

S1 Fig (**A**) Percentage of P-sites in the three frames along the 5’ UTR, CDS and 3’ UTR from ribosome profiling in Hek-293 (Gao et al., 2015). The statistical significances from two-tailed Wilcoxon–Mann–Whitney test comparing RiboProfiling and Plastid with respect to riboWaltz are reported (P-value: *** < 0.001). (**B**) Meta-profiles showing the periodicity of ribosomes along the transcripts at the genome-wide scale. The three metaprofiles are based on the P-site identification obtained by using riboWaltz, RiboProfiling and Plastid. The shaded areas to the left of the start codon highlight the shift of the periodicity toward the 5’ UTR that is absent in the case of data analysed using riboWaltz. (**C**) Comparison between the codon usage index based on in-frame P-sites from riboWaltz and RiboProfiling (left panel) and between the codon usage index based on in-frame P-sites from riboWaltz and Plastid (right panel). The length of the reads ranges from 25 up to 34 nucleotides (see Table 1) with the optimal PO used in the correction step of riboWaltz being 12 nucleotides from the 5’ end.
(TIF)

S2 Fig (**A**) Percentage of P-sites in the three frames along the 5’ UTR, CDS and 3’ UTR from ribosome profiling in MCF-7 (GSE111866). The statistical significances from two-tailed Wilcoxon–Mann–Whitney test comparing RiboProfiling and Plastid with respect to riboWaltz are reported (P-value: * < 0.05, ** < 0.01, *** < 0.001). (**B**) Meta-profiles showing the periodicity of ribosomes along the transcripts at the genome-wide scale. The three metaprofiles are based on the P-site identification obtained by using riboWaltz, RiboProfiling and Plastid. The shaded areas to the left of the start codon highlight the shift of the periodicity toward the 5’ UTR that is absent in the case of data analysed using riboWaltz. (**C**) Comparison between the codon usage index based on in-frame P-sites from riboWaltz and RiboProfiling (left panel) and between the codon usage index based on in-frame P-sites from riboWaltz and Plastid (right panel). The length of the reads ranges from 20 to 45 nucleotides (see S2 Text) with the optimal PO used in the correction step of riboWaltz being 11 nucleotides from the 5’ end.
(TIF)

S3 Fig (**A**) Percentage of P-sites in the three frames along the 5’ UTR, CDS and 3’ UTR from ribosome profiling in mouse after immunoprecipitation of ribosomes using the ribosomal protein RPL10 as tag (Shi et al. 2017). The statistical significances from two-tailed Wilcoxon–Mann–Whitney test comparing RiboProfiling and Plastid with respect to riboWaltz are reported (P-value: * < 0.05, ** < 0.01, *** < 0.001). (**B**) Meta-profiles showing the periodicity of ribosomes along the transcripts at the genome-wide scale. The three metaprofiles are based on the P-site identification obtained by using riboWaltz, RiboProfiling and Plastid. The shaded areas to the left of the start codon highlight the shift of the periodicity toward the 5’ UTR that is absent in the case of data analysed using riboWaltz. (**C**) Comparison between the codon usage index based on in-frame P-sites from riboWaltz and RiboProfiling (left panel) and between the codon usage index based on in-frame P-sites from riboWaltz and Plastid (right panel). The length of the reads ranges from 19 up to 50 nucleotides (see S3 Text) with the optimal PO used in the correction step of riboWaltz being 11 nucleotides from the 5’ end.
(TIF)

S4 Fig (**A**) Percentage of P-sites in the three frames along the 5’ UTR, CDS and 3’ UTR from ribosome profiling in mouse after immunoprecipitation of ribosomes using the ribosomal protein RPL22 as tag (Shi et al. 2017). The statistical significances from two-tailed Wilcoxon–Mann–Whitney test comparing RiboProfiling and Plastid with respect to riboWaltz are reported (P-value: * < 0.05, *** < 0.001). (**B**) Meta-profiles showing the periodicity of ribosomes along the transcripts at the genome-wide scale. The three metaprofiles are based on the P-site identification obtained by using riboWaltz, RiboProfiling and Plastid. The shaded areas to the left of the start codon highlight the shift of the periodicity toward the 5’ UTR that is absent in the case of data analysed using riboWaltz. (**C**) Comparison between the codon usage index based on in-frame P-sites from riboWaltz and RiboProfiling (left panel) and between the codon usage index based on in-frame P-sites from riboWaltz and Plastid (right panel). The length of the reads ranges from 19 up to 50 nucleotides (see S2 Text) with the optimal PO used in the correction step of riboWaltz being 11 nucleotides from the 5’ end.
(TIF)

S5 Fig (**A**) Percentage of P-sites in the three frames along the 5’ UTR, CDS and 3’ UTR from ribosome profiling in yeast (Beaupere et al., 2017). The statistical significances from two-tailed Wilcoxon–Mann–Whitney test comparing RiboProfiling and Plastid with respect to riboWaltz are reported (P-value: * < 0.05, ** < 0.01, *** < 0.001). (**B**) Meta-profiles showing the periodicity of ribosomes along the transcripts at the genome-wide scale. The three metaprofiles are based on the P-site identification obtained by using riboWaltz, RiboProfiling and Plastid. The shaded areas to the left of the start codon highlight the shift of the periodicity toward the 5’ UTR that is absent in the case of data analysed using riboWaltz. (**C**) Comparison between the codon usage index based on in-frame P-sites from riboWaltz and RiboProfiling (left panel) and between the codon usage index based on in-frame P-sites from riboWaltz and Plastid (right panel). The length of the reads ranges from 20 to 46 nucleotides (see S5 Text), with the optimal PO used in the correction step of riboWaltz being 15 nucleotides from the 3’ end.
(TIF)

S6 Fig (**A**) Percentage of P-sites in the three frames along the 5’ UTR, CDS and 3’ UTR from ribosome profiling in yeast (Lareau et al., 2014). The statistical significances from two-tailed Wilcoxon–Mann–Whitney test comparing RiboProfiling and Plastid with respect to riboWaltz are reported (P-value: * < 0.05, *** < 0.001). (**B**) Meta-profiles showing the periodicity of ribosomes along the transcripts at the genome-wide scale. The three metaprofiles are based on the P-site identification obtained by using riboWaltz, RiboProfiling and Plastid. The shaded areas to the left of the start codon highlight the shift of the periodicity toward the 5’ UTR that is absent in the case of data analysed using riboWaltz. (**C**) Comparison between the codon usage index based on in-frame P-sites from riboWaltz and RiboProfiling (left panel) and between the codon usage index based on in-frame P-sites from riboWaltz and Plastid (right panel). The length of the reads ranges from 21 to 40 nucleotides (see S6 Text) with the optimal PO used in the correction step of riboWaltz being 13 nucleotides from the 5’ end.
(TIF)

S7 Fig Performance of riboWaltz compared with RiboProfiling and Plastid in mouse brain tissue (GSE102318) using reads with a length of 27, 28 and 29 nucleotides. (**A**) Percentage of P-sites in the three frames along the 5’ UTR, CDS and 3’ UTR. The statistical significances from two-tailed Wilcoxon–Mann–Whitney test comparing RiboProfiling and Plastid with respect to riboWaltz are reported (P-value: * < 0.05, ** < 0.01, *** < 0.001). (**B**) Meta-profiles showing the periodicity of ribosomes along the transcripts at the genome-wide scale. The three metaprofiles are based on the P-site identification obtained using riboWaltz, RiboProfiling and Plastid. The shaded areas to the left of the start codon highlight the shift of the periodicity toward the 5’ UTR that is absent in the case of data analysed using riboWaltz.
(TIF)

S8 Fig Performance of riboWaltz compared with RiboProfiling and Plastid in Hek-293 cells (Gao et al., 2015) using reads with a length of 27, 28 and 29 nucleotides. (**A**) Percentage of P-sites in the three frames along the 5’ UTR, CDS and 3’ UTR. The statistical significances from two-tailed Wilcoxon–Mann–Whitney test comparing RiboProfiling and Plastid with respect to riboWaltz are reported (P-value: *** < 0.001). (**B**) Meta-profiles showing the periodicity of ribosomes along the transcripts at the genome-wide scale. The three metaprofiles are based on the P-site identification obtained using riboWaltz, RiboProfiling and Plastid. The shaded areas to the left of the start codon highlight the shift of the periodicity toward the 5’ UTR that is absent in the case of data analysed using riboWaltz.
(TIF)

S9 Fig Performance of riboWaltz compared with RiboProfiling and Plastid in MCF-7 cells (GSE111866) using reads with length of 28, 29 and 30 nucleotides. (**A**) Percentage of P-sites in the three frames along the 5’ UTR, CDS and 3’ UTR. The statistical significances from two-tailed Wilcoxon–Mann–Whitney test comparing RiboProfiling and Plastid with respect to riboWaltz are reported (P-value: * < 0.05, *** < 0.001). (**B**) Meta-profiles showing the periodicity of ribosomes along the transcripts at the genome-wide scale. The three metaprofiles are based on the P-site identification obtained using riboWaltz, RiboProfiling and Plastid. The shaded areas to the left of the start codon highlight the shift of the periodicity toward the 5’ UTR that is absent in the case of data analysed using riboWaltz.
(TIF)

S10 Fig Performance of riboWaltz compared with RiboProfiling and Plastid in mouse after immunoprecipitation of ribosomes using the ribosomal protein RPL10 as tag (Shi et al. 2017) using reads with length of 29, 30 and 31 nucleotides. (**A**) Percentage of P-sites in the three frames along the 5’ UTR, CDS and 3’ UTR. The statistical significances from two-tailed Wilcoxon–Mann–Whitney test comparing RiboProfiling and Plastid with respect to riboWaltz are reported (P-value: * < 0.05, *** < 0.001). (**B**) Meta-profiles showing the periodicity of ribosomes along the transcripts at the genome-wide scale. The three metaprofiles are based on the P-site identification obtained using riboWaltz, RiboProfiling and Plastid. The shaded areas to the left of the start codon highlight the shift of the periodicity toward the 5’ UTR that is absent in the case of data analysed using riboWaltz.
(TIF)

S11 Fig Performance of riboWaltz compared with RiboProfiling and Plastid in in mouse after immunoprecipitation of ribosomes using the ribosomal protein RPL22 as tag (Shi et al. 2017) using reads with length of 28, 29 and 30 nucleotides. (**A**) Percentage of P-sites in the three frames along the 5’ UTR, CDS and 3’ UTR. The statistical significances from two-tailed Wilcoxon–Mann–Whitney test comparing RiboProfiling and Plastid with respect to riboWaltz are reported (P-value: *** < 0.001). (**B**) Meta-profiles showing the periodicity of ribosomes along the transcripts at the genome-wide scale. The three metaprofiles are based on the P-site identification obtained using riboWaltz, RiboProfiling and Plastid. The shaded areas to the left of the start codon highlight the shift of the periodicity toward the 5’ UTR that is absent in the case of data analysed using riboWaltz.
(TIF)

S12 Fig Performance of riboWaltz compared with RiboProfiling and Plastid in yeast (Beaupere et al., 2017) using reads with a length of 27, 28 and 29 nucleotides. (**A**) Percentage of P-sites in the three frames along the 5’ UTR, CDS and 3’ UTR. The statistical significances from two-tailed Wilcoxon–Mann–Whitney test comparing RiboProfiling and Plastid with respect to riboWaltz are reported (P-value: ** < 0.01, *** < 0.001). (**B**) Meta-profiles showing the periodicity of ribosomes along the transcripts at the genome-wide scale. The three metaprofiles are based on the P-site identification obtained using riboWaltz, RiboProfiling and Plastid. The shaded areas to the left of the start codon highlight the shift of the periodicity toward the 5’ UTR that is absent in the case of data analysed using riboWaltz.
(TIF)

S13 Fig Performance of riboWaltz compared with RiboProfiling and Plastid in yeast (Lareau et al., 2014) using reads with a length of 28, 29 and 30 nucleotides. (**A**) Percentage of P-sites in the three frames along the 5’ UTR, CDS and 3’ UTR. The statistical significances from two-tailed Wilcoxon–Mann–Whitney test comparing RiboProfiling and Plastid with respect to riboWaltz are reported (P-value: ** < 0.01, *** < 0.001). (**B**) Meta-profiles showing the periodicity of ribosomes along the transcripts at the genome-wide scale. The three metaprofiles are based on the P-site identification obtained using riboWaltz, RiboProfiling and Plastid. The shaded areas to the left of the start codon highlight the shift of the periodicity toward the 5’ UTR that is absent in the case of data analysed using riboWaltz.
(TIF)

S1 Text Comparison of P-site offsets identified for each read length by riboWaltz, RiboProfiling and Plastid in human (Hek-293, Gao et al., 2015). The PO computed from both read extremities are reported. The optimal PO used in the correction step of riboWaltz corresponds to 12 nucleotides from the 5’ end.
(DOCX)

S2 Text Comparison of P-site offsets identified for each read length by riboWaltz, RiboProfiling and Plastid in human (MCF-7, GSE111866). The PO computed from both read extremities are reported. The optimal PO used in the correction step of riboWaltz corresponds to 11 nucleotides from the 5’ end.
(DOCX)

S3 Text Comparison of P-site offsets identified for each read length by riboWaltz, RiboProfiling and Plastid in mouse (after pull-down of RLP10, Shi et al. 2017). The PO computed from both read extremities are reported. The optimal PO used in the correction step of riboWaltz corresponds to 11 nucleotides from the 5’ end.
(DOCX)

S4 Text Comparison of P-site offsets identified for each read length by riboWaltz, RiboProfiling and Plastid in mouse (after pull-down of RLP22, Shi et al. 2017). The PO computed from both read extremities are reported. The optimal PO used in the correction step of riboWaltz corresponds to 11 nucleotides from the 5’ end.
(DOCX)

S5 Text Comparison of P-site offsets identified for each read length by riboWaltz, RiboProfiling and Plastid in yeast (Beaupere et al., 2017). The PO computed from both read extremities are reported. The optimal PO used in the correction step of riboWaltz corresponds to 15 nucleotides from the 3’ end.
(DOCX)

S6 Text Comparison of P-site offsets identified for each read length by riboWaltz, RiboProfiling and Plastid in yeast (Lareau et al., 2014). The PO computed from both read extremities are reported. The optimal PO used in the correction step of riboWaltz corresponds to 13 nucleotides from the 5’ end.
(DOCX)

S7 Text Comparison between temporary and corrected P-site offsets identified by riboWaltz in human (Hek-293, Gao et al., 2015). The PO computed from both read extremities are reported. The optimal PO used in the correction step corresponds to 12 nucleotides from the 5’ end.
(DOCX)

S8 Text Comparison between temporary and corrected P-site offsets identified by riboWaltz in human (MCF-7, GEO111866). The PO computed from both read extremities are reported. The optimal PO used in the correction step corresponds to 11 nucleotides from the 5’ end.
(DOCX)

S9 Text Comparison between temporary and corrected P-site offsets identified by riboWaltz in mouse (after pull-down of RLP10, Shi et al. 2017). The PO computed from both read extremities are reported. The optimal PO used in the correction step corresponds to 11 nucleotides from the 5’ end.
(DOCX)

S10 Text Comparison between temporary and corrected P-site offsets identified by riboWaltz in mouse (after pull-down of RLP22, Shi et al. 2017). The PO computed from both read extremities are reported. The optimal PO used in the correction step corresponds to 11 nucleotides from the 5’ end.
(DOCX)

S11 Text Comparison between temporary and corrected P-site offsets identified by riboWaltz in yeast (Beaupere et al., 2017). The PO computed from both read extremities are reported. The optimal PO used in the correction step corresponds to 15 nucleotides from the 3’ end.
(DOCX)

S12 Text Comparison between temporary and corrected P-site offsets identified by riboWaltz in yeast (Lareau et al., 2014). The PO computed from both read extremities are reported. The optimal PO used in the correction step corresponds to 13 nucleotides from the 5’ end.
(DOCX)

S13 Text Supplementary methods. (DOCX)
