# Supplementary figures and images for: riboWaltz: Optimization of ribosome P-site positioning in ribosome profiling data
Source: PLoS Comput Biol. 2018 Aug 13;14(8):e1006169. doi: 10.1371/journal.pcbi.1006169 (PMC6112680; doi:10.1371/journal.pcbi.1006169)

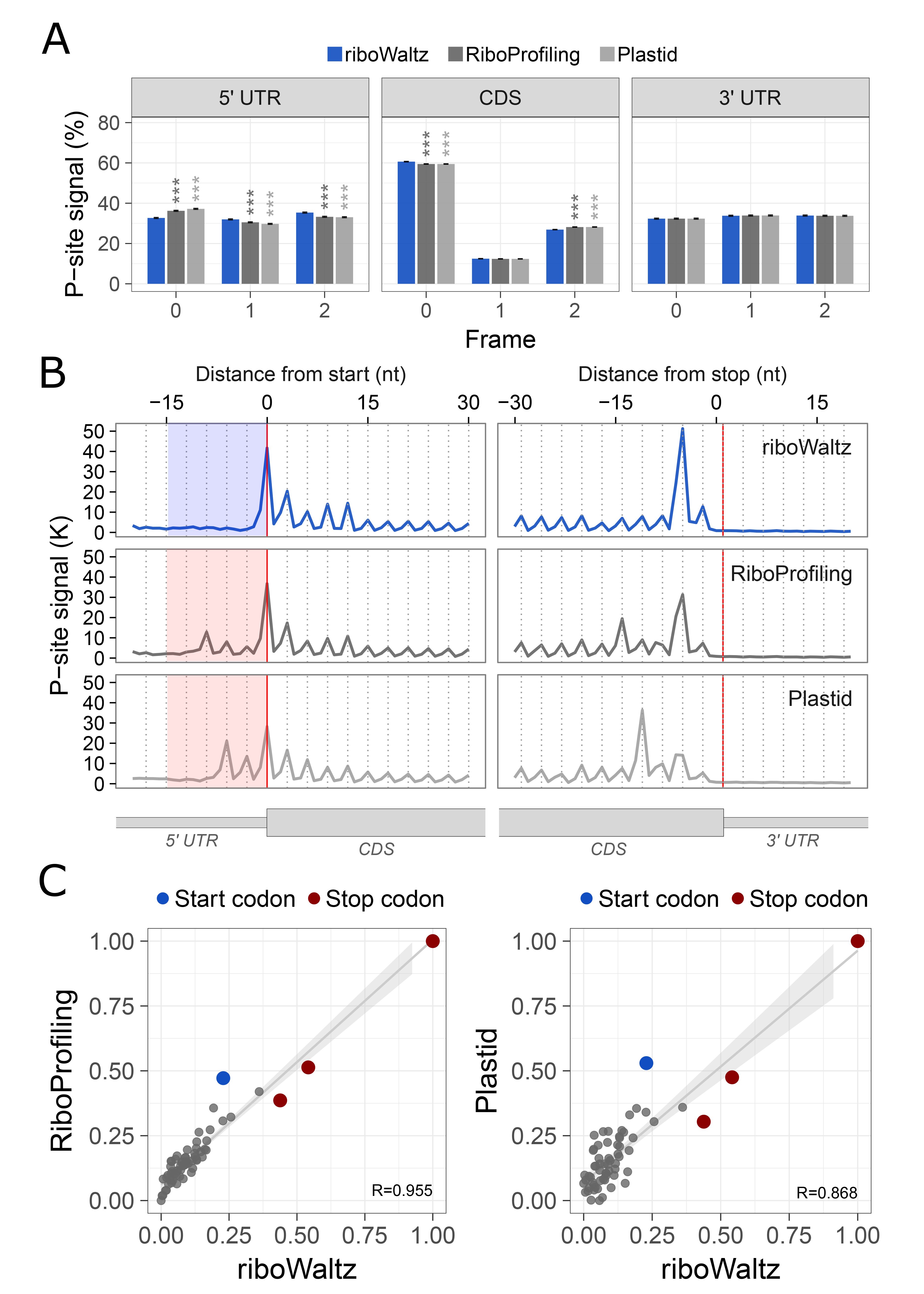

Supplement: S1 Fig — (A) Percentage of P-sites in the three frames along the 5’ UTR, CDS and 3’ UTR from ribosome profiling in Hek-293 (Gao et al., 2015). The statistical significances from two-tailed Wilcoxon–Mann–Whitney test comparing RiboProfiling and Plastid with respect to riboWaltz are reported (P-value: *** < 0.001). (B) Meta-profiles showing the periodicity of ribosomes along the transcripts at the genome-wide scale. The three metaprofiles are based on the P-site identification obtained by using riboWaltz, RiboProfiling and Plastid. The shaded areas to the left of the start codon highlight the shift of the periodicity toward the 5’ UTR that is absent in the case of data analysed using riboWaltz. (C) Comparison between the codon usage index based on in-frame P-sites from riboWaltz and RiboProfiling (left panel) and between the codon usage index based on in-frame P-sites from riboWaltz and Plastid (right panel). The length of the reads ranges from 25 up to 34 nucleotides (see Table 1) with the optimal PO used in the correction step of riboWaltz being 12 nucleotides from the 5’ end. (TIF) [file pcbi.1006169.s001.tif]

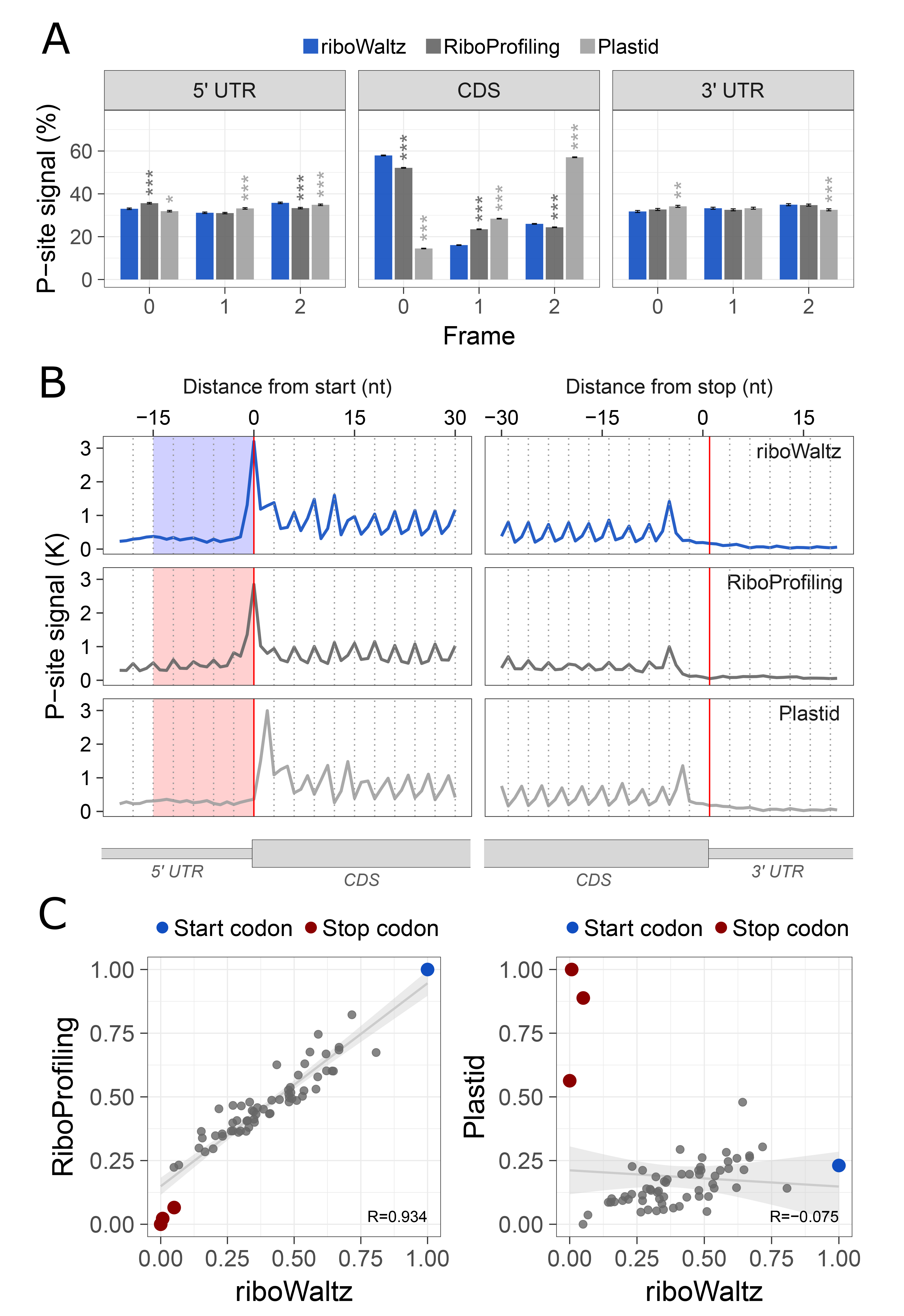

Supplement: S2 Fig — (A) Percentage of P-sites in the three frames along the 5’ UTR, CDS and 3’ UTR from ribosome profiling in MCF-7 (GSE111866). The statistical significances from two-tailed Wilcoxon–Mann–Whitney test comparing RiboProfiling and Plastid with respect to riboWaltz are reported (P-value: * < 0.05, ** < 0.01, *** < 0.001). (B) Meta-profiles showing the periodicity of ribosomes along the transcripts at the genome-wide scale. The three metaprofiles are based on the P-site identification obtained by using riboWaltz, RiboProfiling and Plastid. The shaded areas to the left of the start codon highlight the shift of the periodicity toward the 5’ UTR that is absent in the case of data analysed using riboWaltz. (C) Comparison between the codon usage index based on in-frame P-sites from riboWaltz and RiboProfiling (left panel) and between the codon usage index based on in-frame P-sites from riboWaltz and Plastid (right panel). The length of the reads ranges from 20 to 45 nucleotides (see S2 Text) with the optimal PO used in the correction step of riboWaltz being 11 nucleotides from the 5’ end. (TIF) [file pcbi.1006169.s002.tif]

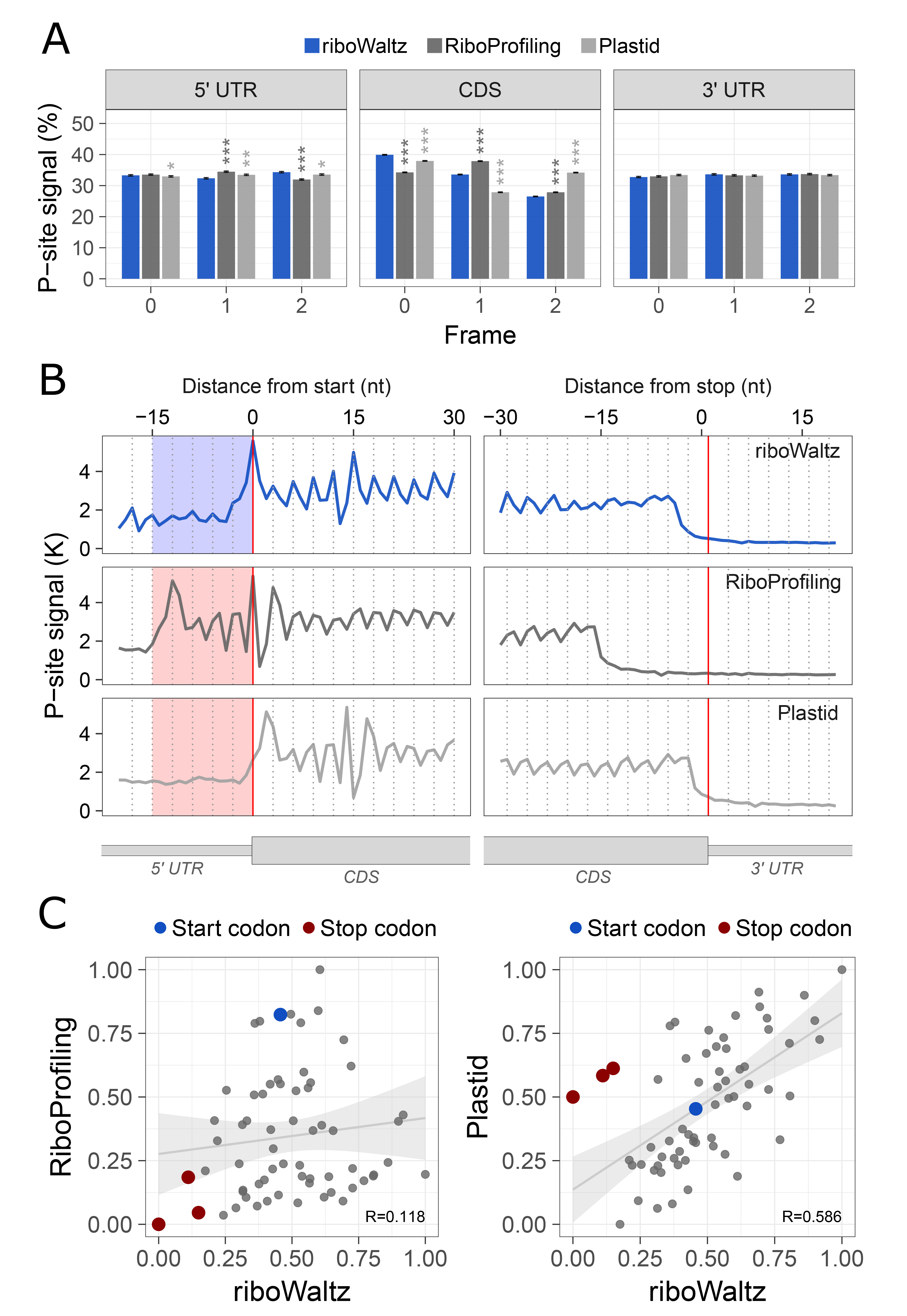

Supplement: S3 Fig — (A) Percentage of P-sites in the three frames along the 5’ UTR, CDS and 3’ UTR from ribosome profiling in mouse after immunoprecipitation of ribosomes using the ribosomal protein RPL10 as tag (Shi et al. 2017). The statistical significances from two-tailed Wilcoxon–Mann–Whitney test comparing RiboProfiling and Plastid with respect to riboWaltz are reported (P-value: * < 0.05, ** < 0.01, *** < 0.001). (B) Meta-profiles showing the periodicity of ribosomes along the transcripts at the genome-wide scale. The three metaprofiles are based on the P-site identification obtained by using riboWaltz, RiboProfiling and Plastid. The shaded areas to the left of the start codon highlight the shift of the periodicity toward the 5’ UTR that is absent in the case of data analysed using riboWaltz. (C) Comparison between the codon usage index based on in-frame P-sites from riboWaltz and RiboProfiling (left panel) and between the codon usage index based on in-frame P-sites from riboWaltz and Plastid (right panel). The length of the reads ranges from 19 up to 50 nucleotides (see S3 Text) with the optimal PO used in the correction step of riboWaltz being 11 nucleotides from the 5’ end. (TIF) [file pcbi.1006169.s003.tif]

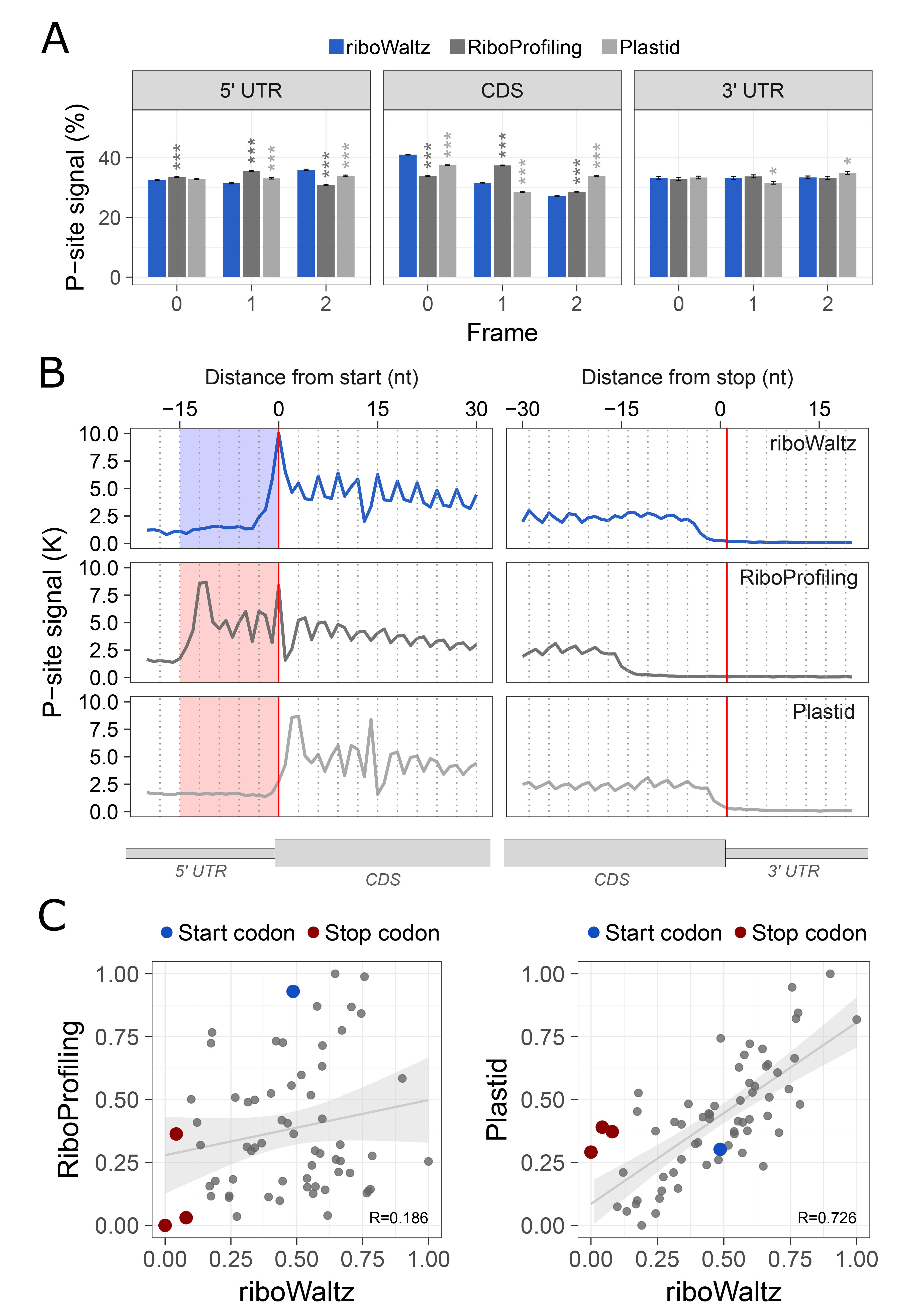

Supplement: S4 Fig — (A) Percentage of P-sites in the three frames along the 5’ UTR, CDS and 3’ UTR from ribosome profiling in mouse after immunoprecipitation of ribosomes using the ribosomal protein RPL22 as tag (Shi et al. 2017). The statistical significances from two-tailed Wilcoxon–Mann–Whitney test comparing RiboProfiling and Plastid with respect to riboWaltz are reported (P-value: * < 0.05, *** < 0.001). (B) Meta-profiles showing the periodicity of ribosomes along the transcripts at the genome-wide scale. The three metaprofiles are based on the P-site identification obtained by using riboWaltz, RiboProfiling and Plastid. The shaded areas to the left of the start codon highlight the shift of the periodicity toward the 5’ UTR that is absent in the case of data analysed using riboWaltz. (C) Comparison between the codon usage index based on in-frame P-sites from riboWaltz and RiboProfiling (left panel) and between the codon usage index based on in-frame P-sites from riboWaltz and Plastid (right panel). The length of the reads ranges from 19 up to 50 nucleotides (see S2 Text) with the optimal PO used in the correction step of riboWaltz being 11 nucleotides from the 5’ end. (TIF) [file pcbi.1006169.s004.tif]

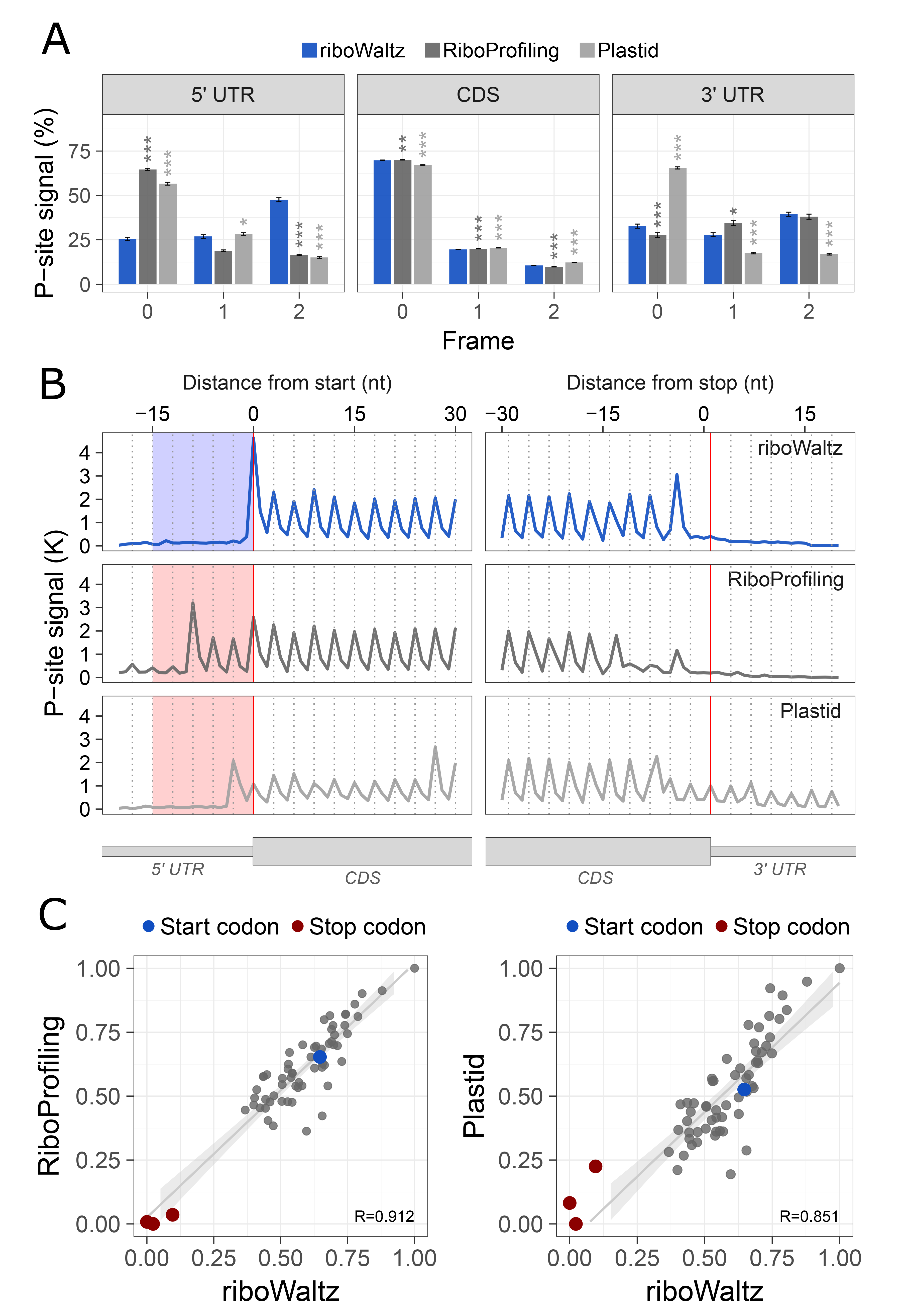

Supplement: S5 Fig — (A) Percentage of P-sites in the three frames along the 5’ UTR, CDS and 3’ UTR from ribosome profiling in yeast (Beaupere et al., 2017). The statistical significances from two-tailed Wilcoxon–Mann–Whitney test comparing RiboProfiling and Plastid with respect to riboWaltz are reported (P-value: * < 0.05, ** < 0.01, *** < 0.001). (B) Meta-profiles showing the periodicity of ribosomes along the transcripts at the genome-wide scale. The three metaprofiles are based on the P-site identification obtained by using riboWaltz, RiboProfiling and Plastid. The shaded areas to the left of the start codon highlight the shift of the periodicity toward the 5’ UTR that is absent in the case of data analysed using riboWaltz. (C) Comparison between the codon usage index based on in-frame P-sites from riboWaltz and RiboProfiling (left panel) and between the codon usage index based on in-frame P-sites from riboWaltz and Plastid (right panel). The length of the reads ranges from 20 to 46 nucleotides (see S5 Text), with the optimal PO used in the correction step of riboWaltz being 15 nucleotides from the 3’ end. (TIF) [file pcbi.1006169.s005.tif]

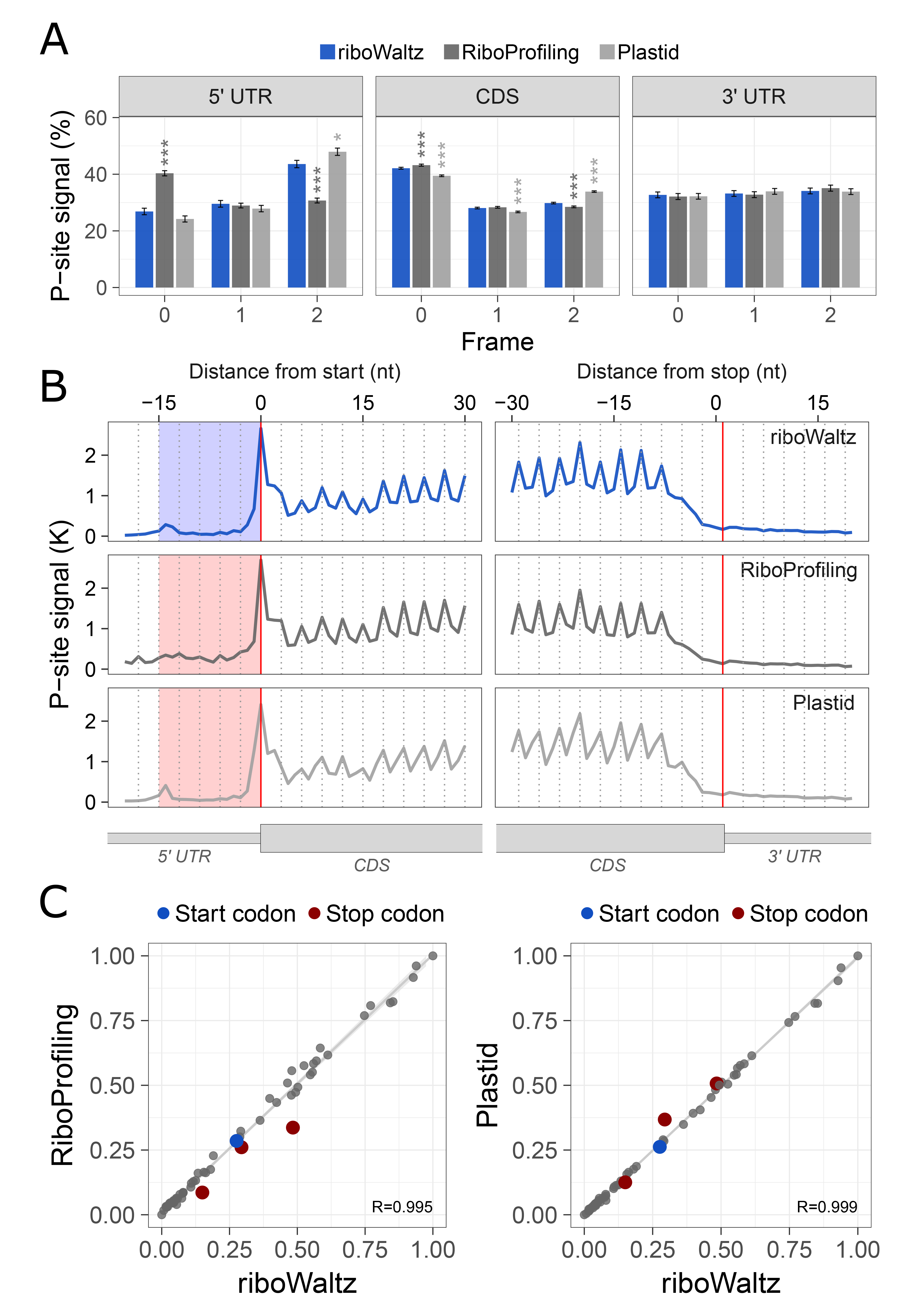

Supplement: S6 Fig — (A) Percentage of P-sites in the three frames along the 5’ UTR, CDS and 3’ UTR from ribosome profiling in yeast (Lareau et al., 2014). The statistical significances from two-tailed Wilcoxon–Mann–Whitney test comparing RiboProfiling and Plastid with respect to riboWaltz are reported (P-value: * < 0.05, *** < 0.001). (B) Meta-profiles showing the periodicity of ribosomes along the transcripts at the genome-wide scale. The three metaprofiles are based on the P-site identification obtained by using riboWaltz, RiboProfiling and Plastid. The shaded areas to the left of the start codon highlight the shift of the periodicity toward the 5’ UTR that is absent in the case of data analysed using riboWaltz. (C) Comparison between the codon usage index based on in-frame P-sites from riboWaltz and RiboProfiling (left panel) and between the codon usage index based on in-frame P-sites from riboWaltz and Plastid (right panel). The length of the reads ranges from 21 to 40 nucleotides (see S6 Text) with the optimal PO used in the correction step of riboWaltz being 13 nucleotides from the 5’ end. (TIF) [file pcbi.1006169.s006.tif]

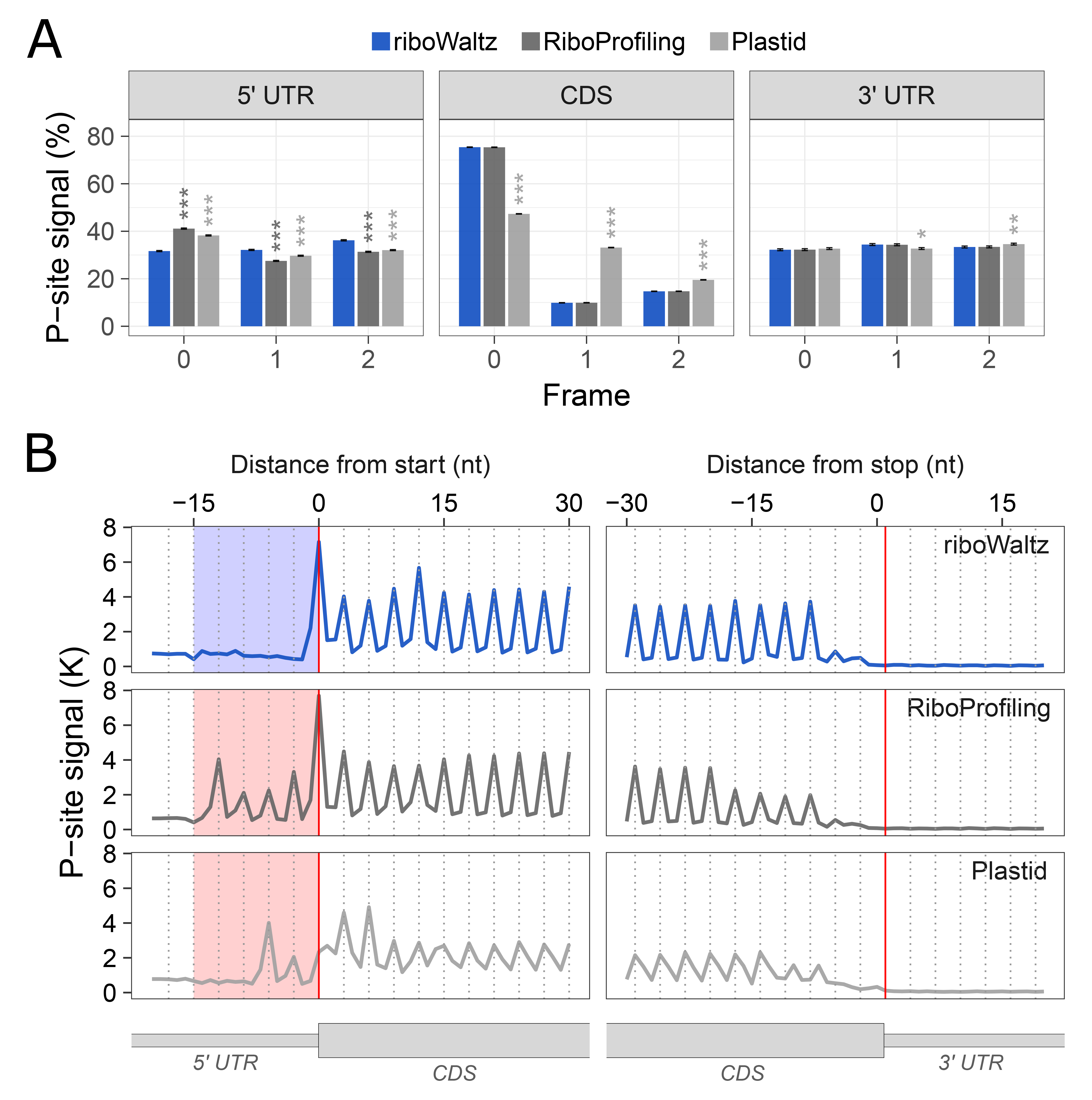

Supplement: S7 Fig — (A) Percentage of P-sites in the three frames along the 5’ UTR, CDS and 3’ UTR. The statistical significances from two-tailed Wilcoxon–Mann–Whitney test comparing RiboProfiling and Plastid with respect to riboWaltz are reported (P-value: * < 0.05, ** < 0.01, *** < 0.001). (B) Meta-profiles showing the periodicity of ribosomes along the transcripts at the genome-wide scale. The three metaprofiles are based on the P-site identification obtained using riboWaltz, RiboProfiling and Plastid. The shaded areas to the left of the start codon highlight the shift of the periodicity toward the 5’ UTR that is absent in the case of data analysed using riboWaltz. (TIF) [file pcbi.1006169.s007.tif]

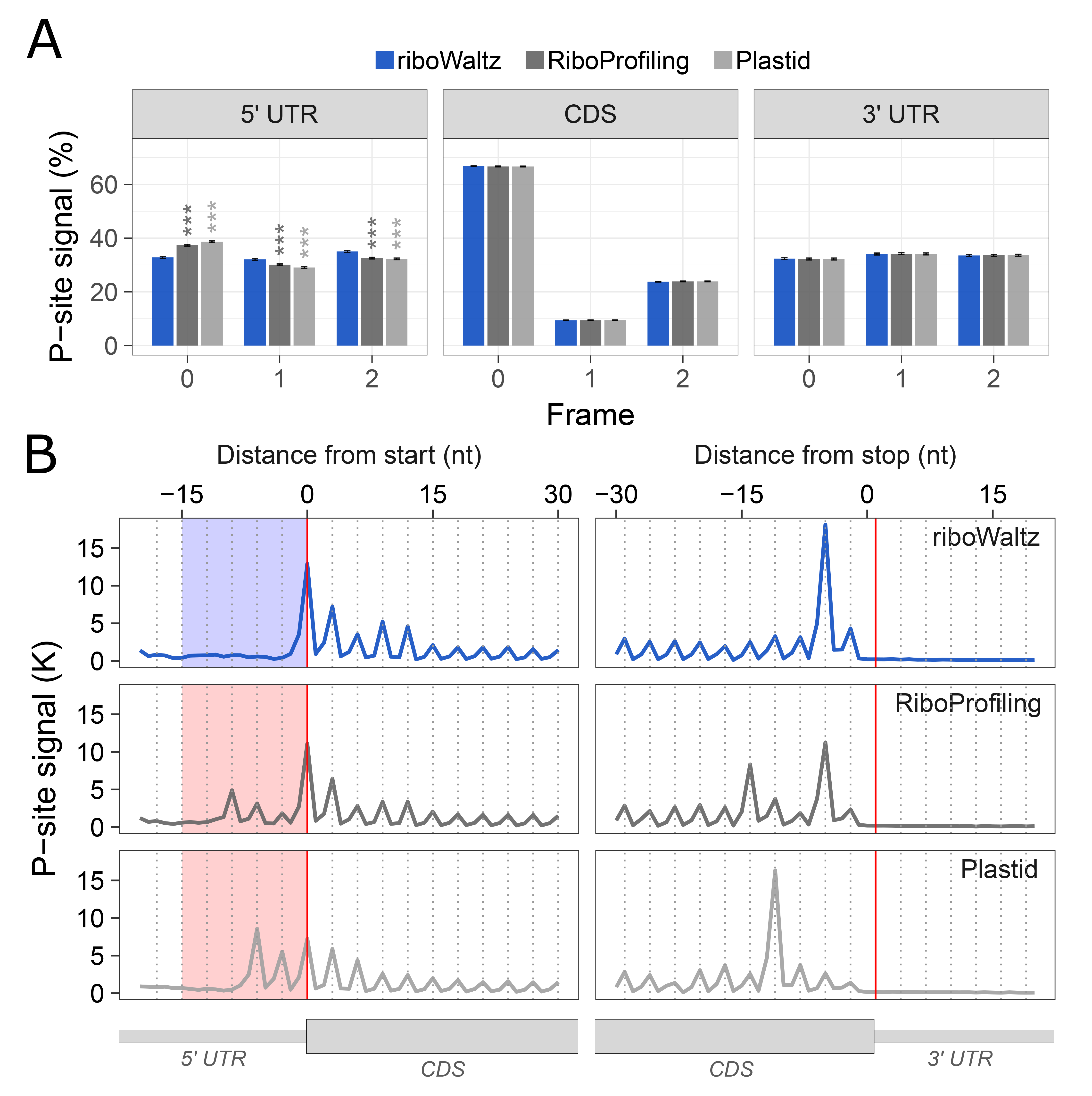

Supplement: S8 Fig — (A) Percentage of P-sites in the three frames along the 5’ UTR, CDS and 3’ UTR. The statistical significances from two-tailed Wilcoxon–Mann–Whitney test comparing RiboProfiling and Plastid with respect to riboWaltz are reported (P-value: *** < 0.001). (B) Meta-profiles showing the periodicity of ribosomes along the transcripts at the genome-wide scale. The three metaprofiles are based on the P-site identification obtained using riboWaltz, RiboProfiling and Plastid. The shaded areas to the left of the start codon highlight the shift of the periodicity toward the 5’ UTR that is absent in the case of data analysed using riboWaltz. (TIF) [file pcbi.1006169.s008.tif]

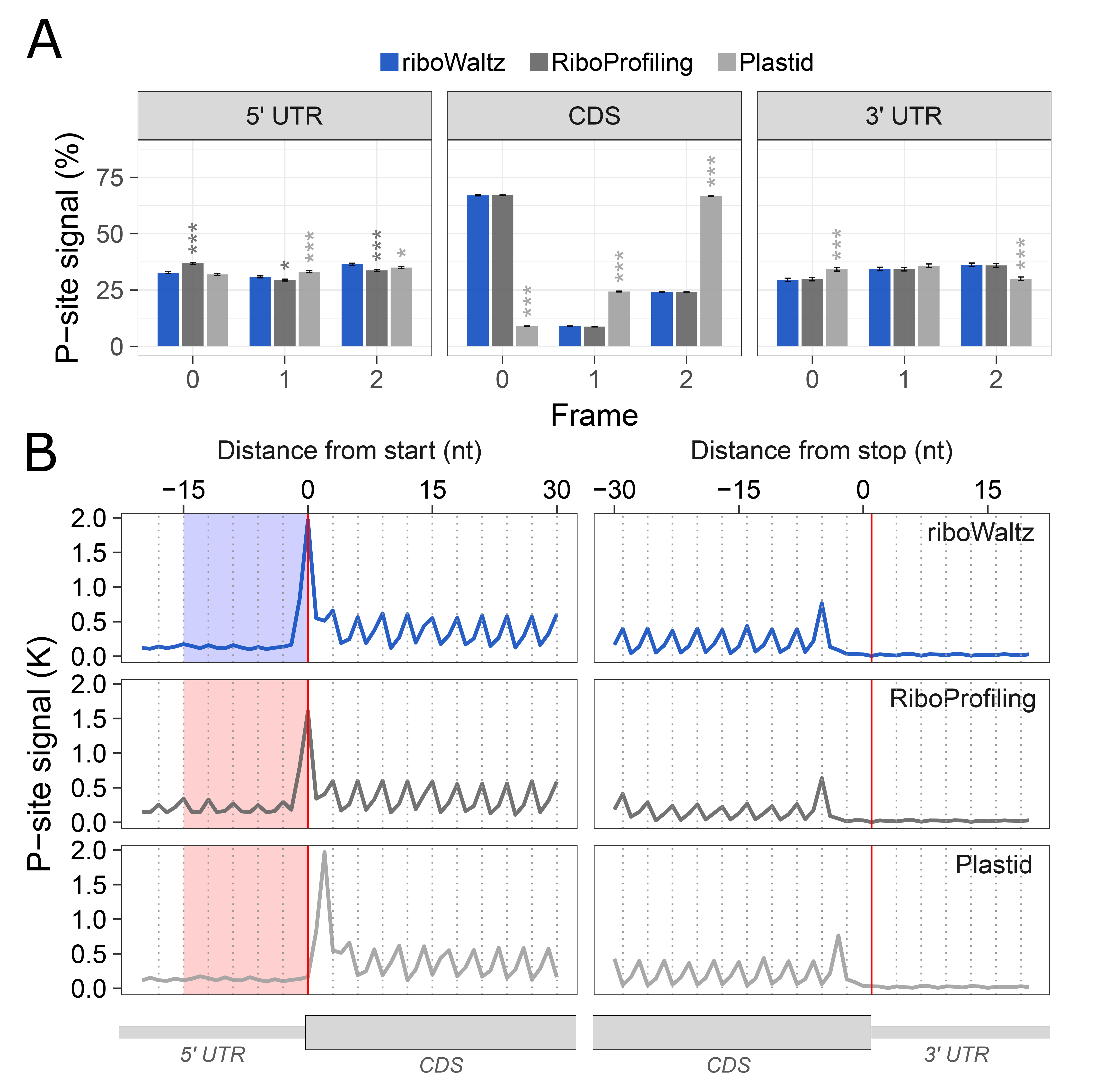

Supplement: S9 Fig — (A) Percentage of P-sites in the three frames along the 5’ UTR, CDS and 3’ UTR. The statistical significances from two-tailed Wilcoxon–Mann–Whitney test comparing RiboProfiling and Plastid with respect to riboWaltz are reported (P-value: * < 0.05, *** < 0.001). (B) Meta-profiles showing the periodicity of ribosomes along the transcripts at the genome-wide scale. The three metaprofiles are based on the P-site identification obtained using riboWaltz, RiboProfiling and Plastid. The shaded areas to the left of the start codon highlight the shift of the periodicity toward the 5’ UTR that is absent in the case of data analysed using riboWaltz. (TIF) [file pcbi.1006169.s009.tif]

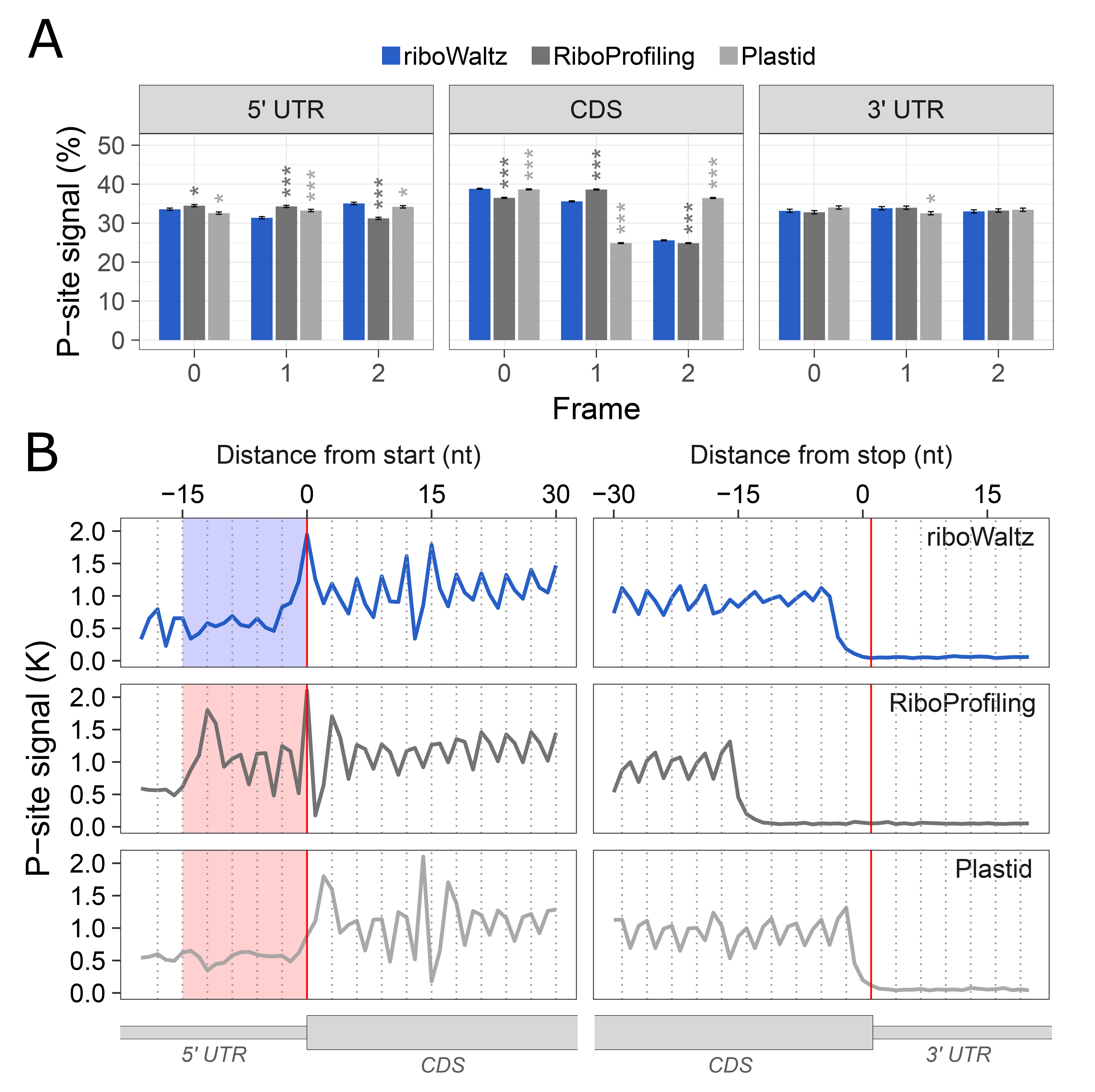

Supplement: S10 Fig — (A) Percentage of P-sites in the three frames along the 5’ UTR, CDS and 3’ UTR. The statistical significances from two-tailed Wilcoxon–Mann–Whitney test comparing RiboProfiling and Plastid with respect to riboWaltz are reported (P-value: * < 0.05, *** < 0.001). (B) Meta-profiles showing the periodicity of ribosomes along the transcripts at the genome-wide scale. The three metaprofiles are based on the P-site identification obtained using riboWaltz, RiboProfiling and Plastid. The shaded areas to the left of the start codon highlight the shift of the periodicity toward the 5’ UTR that is absent in the case of data analysed using riboWaltz. (TIF) [file pcbi.1006169.s010.tif]

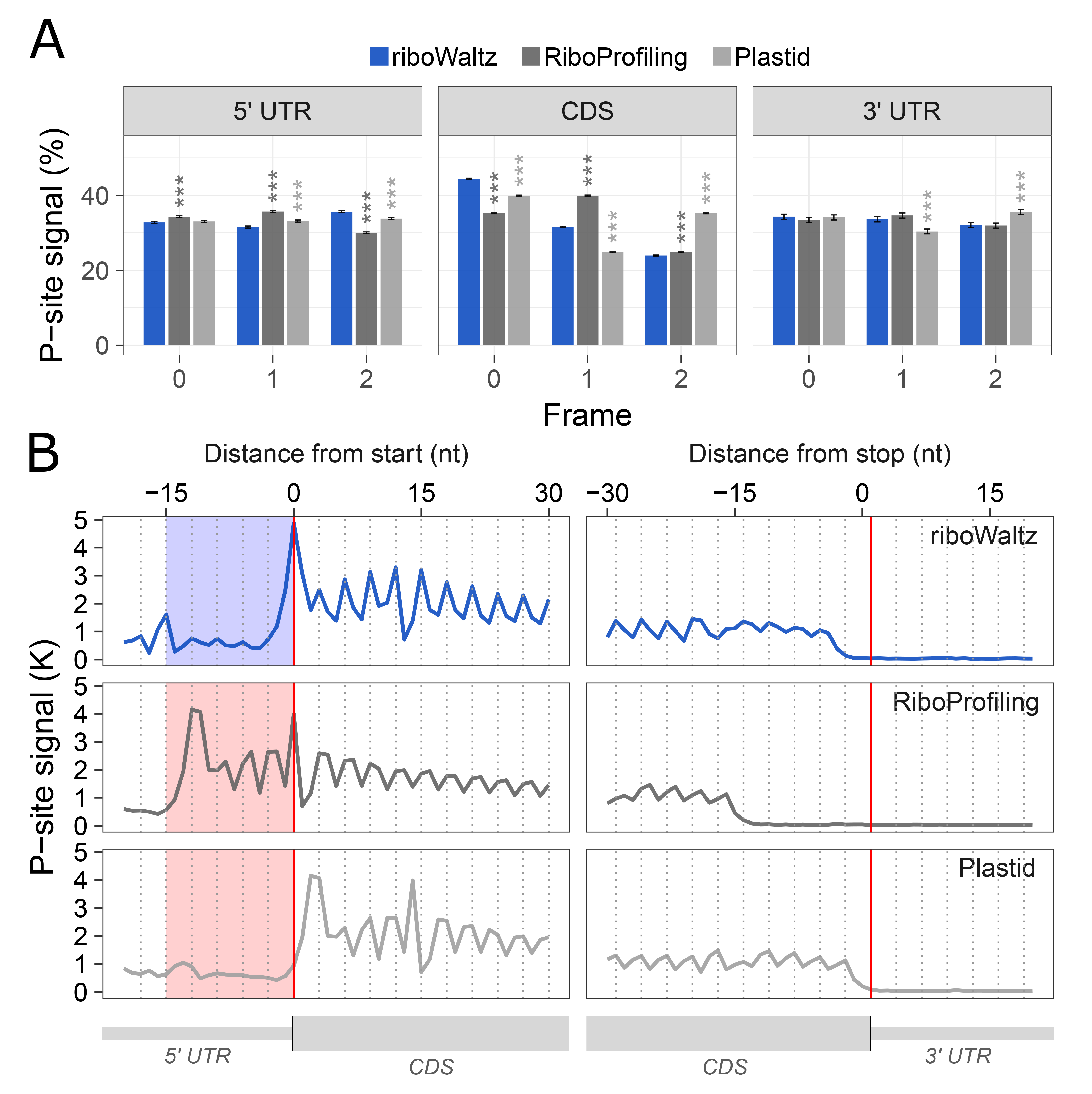

Supplement: S11 Fig — (A) Percentage of P-sites in the three frames along the 5’ UTR, CDS and 3’ UTR. The statistical significances from two-tailed Wilcoxon–Mann–Whitney test comparing RiboProfiling and Plastid with respect to riboWaltz are reported (P-value: *** < 0.001). (B) Meta-profiles showing the periodicity of ribosomes along the transcripts at the genome-wide scale. The three metaprofiles are based on the P-site identification obtained using riboWaltz, RiboProfiling and Plastid. The shaded areas to the left of the start codon highlight the shift of the periodicity toward the 5’ UTR that is absent in the case of data analysed using riboWaltz. (TIF) [file pcbi.1006169.s011.tif]

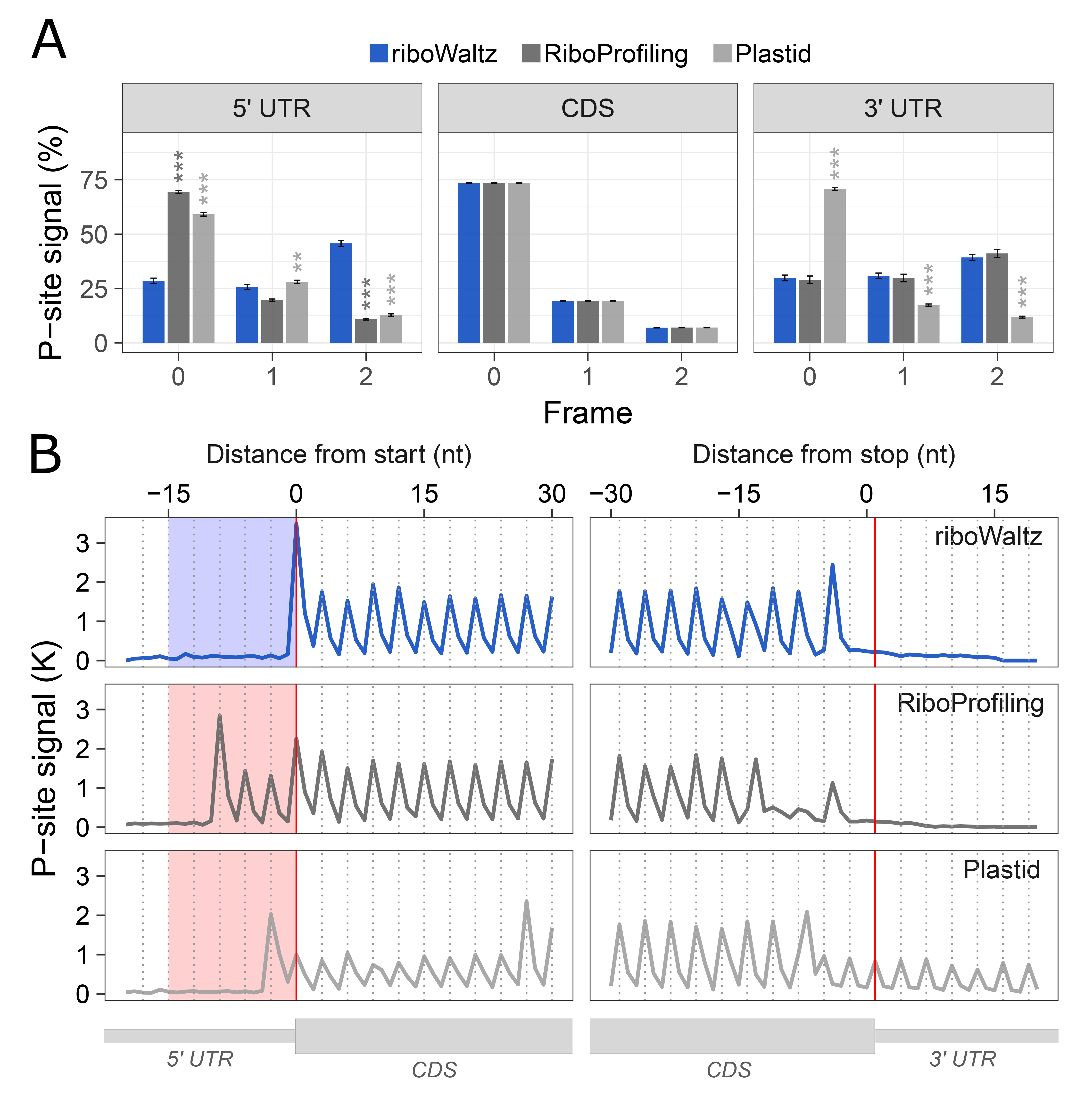

Supplement: S12 Fig — (A) Percentage of P-sites in the three frames along the 5’ UTR, CDS and 3’ UTR. The statistical significances from two-tailed Wilcoxon–Mann–Whitney test comparing RiboProfiling and Plastid with respect to riboWaltz are reported (P-value: ** < 0.01, *** < 0.001). (B) Meta-profiles showing the periodicity of ribosomes along the transcripts at the genome-wide scale. The three metaprofiles are based on the P-site identification obtained using riboWaltz, RiboProfiling and Plastid. The shaded areas to the left of the start codon highlight the shift of the periodicity toward the 5’ UTR that is absent in the case of data analysed using riboWaltz. (TIF) [file pcbi.1006169.s012.tif]

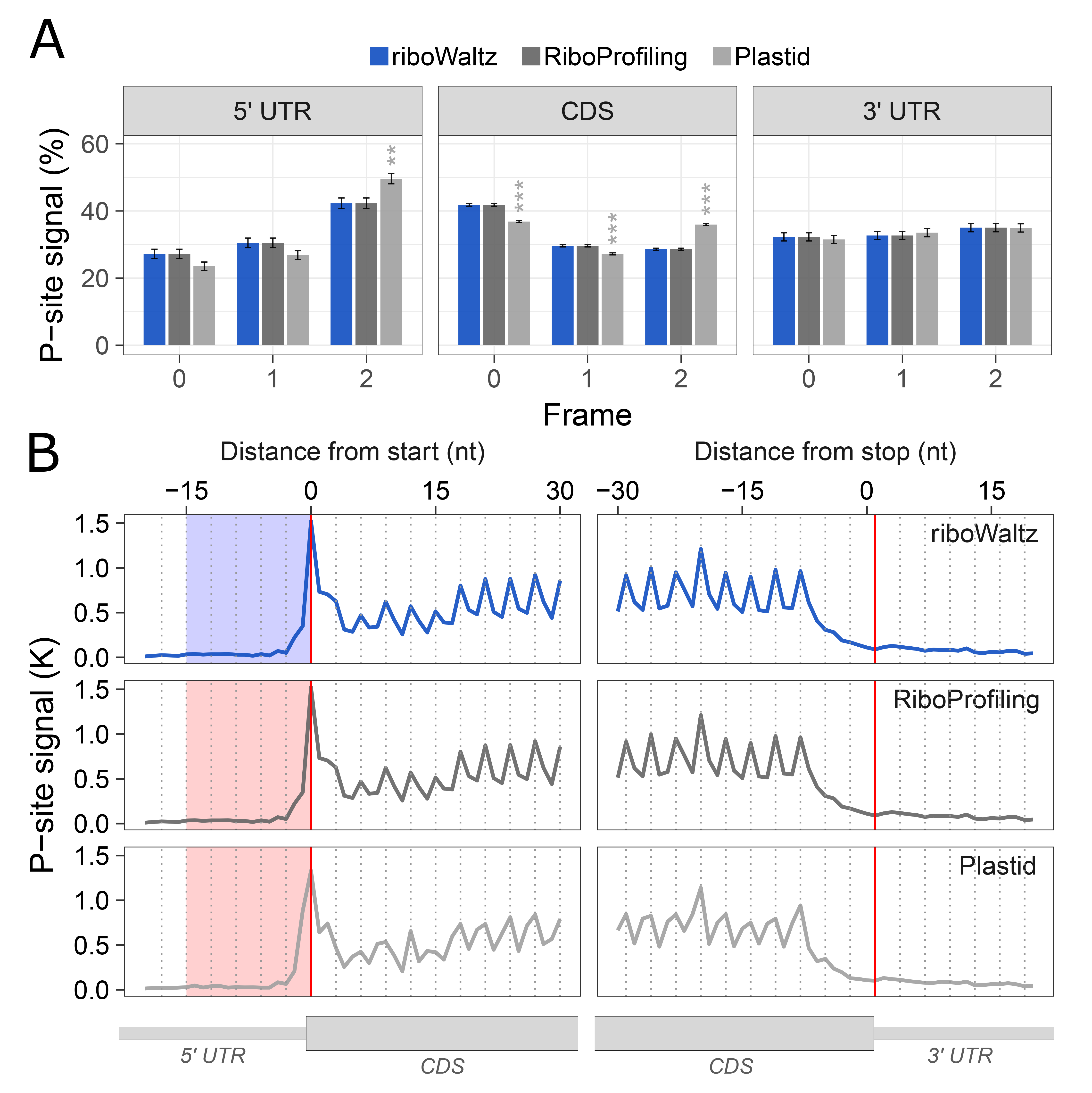

Supplement: S13 Fig — (A) Percentage of P-sites in the three frames along the 5’ UTR, CDS and 3’ UTR. The statistical significances from two-tailed Wilcoxon–Mann–Whitney test comparing RiboProfiling and Plastid with respect to riboWaltz are reported (P-value: ** < 0.01, *** < 0.001). (B) Meta-profiles showing the periodicity of ribosomes along the transcripts at the genome-wide scale. The three metaprofiles are based on the P-site identification obtained using riboWaltz, RiboProfiling and Plastid. The shaded areas to the left of the start codon highlight the shift of the periodicity toward the 5’ UTR that is absent in the case of data analysed using riboWaltz. (TIF) [file pcbi.1006169.s013.tif]
