# Supplementary material for: riboWaltz: Optimization of ribosome P-site positioning in ribosome profiling data
Source: PLoS Comput Biol. 2018 Aug 13;14(8):e1006169. doi: 10.1371/journal.pcbi.1006169 (PMC6112680; doi:10.1371/journal.pcbi.1006169)
Supplement: S1 Text — The PO computed from both read extremities are reported. The optimal PO used in the correction step of riboWaltz corresponds to 12 nucleotides from the 5’ end. (DOCX) [file pcbi.1006169.s014.docx]

| **Read length** | **riboWaltz** | | **RiboProfiling** | | **Plastid** | |
| --- | --- | --- | --- | --- | --- | --- |
|  | from 5’ end | from 3’ end | from 5’ end | from 3’ end | from 5’ end | from 3’ end |
| **25** | 12 | 12 | 0 | 24 | 3 | 21 |
| **26** | 12 | 13 | 12 | 13 | 12 | 13 |
| **27** | 12 | 14 | 12 | 14 | 12 | 14 |
| **28** | 12 | 15 | 3 | 24 | 6 | 21 |
| **29** | 12 | 16 | 12 | 16 | 6 | 22 |
| **30** | 12 | 17 | 12 | 17 | 12 | 17 |
| **31** | 13 | 17 | 9 | 21 | 9 | 21 |
| **32** | 13 | 18 | 10 | 21 | 7 | 24 |
| **33** | 12 | 20 | 12 | 20 | 50 | 18 |
| **34** | 15 | 18 | 17 | 16 | 13 | 20 |
