# Supplementary material for: riboWaltz: Optimization of ribosome P-site positioning in ribosome profiling data
Source: PLoS Comput Biol. 2018 Aug 13;14(8):e1006169. doi: 10.1371/journal.pcbi.1006169 (PMC6112680; doi:10.1371/journal.pcbi.1006169)
Supplement: S2 Text — The PO computed from both read extremities are reported. The optimal PO used in the correction step of riboWaltz corresponds to 11 nucleotides from the 5’ end. (DOCX) [file pcbi.1006169.s015.docx]

| **Read length** | **riboWaltz** | | **RiboProfiling** | | **Plastid** | |
| --- | --- | --- | --- | --- | --- | --- |
|  | from 5’ end | from 3’ end | from 5’ end | from 3’ end | from 5’ end | from 3’ end |
| **20** | 11 | 8 | -1 | 20 | 13 | 6 |
| **21** | 11 | 9 | 3 | 17 | 13 | 7 |
| **22** | 11 | 10 | 4 | 17 | 13 | 8 |
| **23** | 11 | 11 | 23 | -1 | 13 | 9 |
| **24** | 11 | 12 | 6 | 17 | 13 | 10 |
| **25** | 11 | 13 | 25 | -1 | 13 | 11 |
| **26** | 11 | 14 | 9 | 16 | 13 | 12 |
| **27** | 11 | 15 | 9 | 17 | 13 | 13 |
| **28** | 11 | 16 | -13 | 40 | 13 | 14 |
| **29** | 11 | 17 | 11 | 17 | 13 | 15 |
| **30** | 11 | 18 | 11 | 18 | 13 | 16 |
| **31** | 12 | 18 | 12 | 18 | 13 | 17 |
| **32** | 12 | 19 | 12 | 19 | 13 | 18 |
| **33** | 12 | 20 | -10 | 42 | 13 | 19 |
| **34** | 11 | 22 | 17 | 16 | 13 | 20 |
| **35** | 10 | 24 | 4 | 30 | 13 | 21 |
| **36** | 12 | 23 | 12 | 23 | 13 | 22 |
| **37** | 10 | 26 | 35 | 1 | 13 | 23 |
| **38** | 12 | 25 | -7 | 44 | 13 | 24 |
| **39** | 10 | 28 | 20 | 18 | 13 | 25 |
| **41** | 23 | 17 | -14 | 54 | 13 | 27 |
| **42** | 17 | 24 | 37 | 4 | 13 | 28 |
| **43** | 11 | 31 | 0 | 42 | 13 | 29 |
| **45** | 14 | 30 | 48 | -4 | 13 | 31 |
