# Supplementary material for: riboWaltz: Optimization of ribosome P-site positioning in ribosome profiling data
Source: PLoS Comput Biol. 2018 Aug 13;14(8):e1006169. doi: 10.1371/journal.pcbi.1006169 (PMC6112680; doi:10.1371/journal.pcbi.1006169)
Supplement: S4 Text — The PO computed from both read extremities are reported. The optimal PO used in the correction step of riboWaltz corresponds to 11 nucleotides from the 5’ end. (DOCX) [file pcbi.1006169.s017.docx]

| **Read length** | **riboWaltz** | | **RiboProfiling** | | **Plastid** | |
| --- | --- | --- | --- | --- | --- | --- |
|  | from 5’ end | from 3’ end | from 5’ end | from 3’ end | from 5’ end | from 3’ end |
| **19** | 12 | 6 | -1 | 19 | 13 | 5 |
| **20** | 11 | 8 | -1 | 20 | 13 | 6 |
| **21** | 12 | 8 | -1 | 21 | 13 | 7 |
| **22** | 11 | 10 | -1 | 22 | 13 | 8 |
| **23** | 10 | 12 | -1 | 23 | 13 | 9 |
| **24** | 9 | 14 | -1 | 24 | 13 | 10 |
| **25** | 10 | 14 | -1 | 25 | 13 | 11 |
| **26** | 10 | 15 | -1 | 26 | 13 | 12 |
| **27** | 11 | 15 | -1 | 27 | 13 | 13 |
| **28** | 10 | 17 | -1 | 28 | 13 | 14 |
| **29** | 11 | 17 | -1 | 29 | 13 | 15 |
| **30** | 11 | 18 | -1 | 30 | 13 | 16 |
| **31** | 10 | 20 | -1 | 31 | 13 | 17 |
| **32** | 11 | 20 | -1 | 32 | 13 | 18 |
| **33** | 12 | 20 | -1 | 33 | 13 | 19 |
| **34** | 10 | 23 | -1 | 34 | 13 | 20 |
| **35** | 10 | 24 | -1 | 35 | 13 | 21 |
| **36** | 10 | 25 | -1 | 36 | 13 | 22 |
| **37** | 10 | 26 | -1 | 37 | 13 | 23 |
| **38** | 10 | 27 | -1 | 38 | 13 | 24 |
| **39** | 11 | 27 | -1 | 39 | 13 | 25 |
| **40** | 10 | 29 | -1 | 40 | 13 | 26 |
| **41** | 11 | 29 | -1 | 41 | 13 | 27 |
| **42** | 11 | 30 | -1 | 42 | 13 | 28 |
| **43** | 7 | 35 | -1 | 43 | 13 | 29 |
| **44** | 10 | 33 | -1 | 44 | 13 | 30 |
| **45** | 16 | 28 | -1 | 45 | 13 | 31 |
| **46** | 11 | 34 | -1 | 46 | 13 | 32 |
| **47** | 11 | 35 | -1 | 47 | 13 | 33 |
| **48** | 11 | 36 | -1 | 48 | 13 | 34 |
| **49** | 11 | 37 | -1 | 49 | 13 | 35 |
| **50** | 11 | 38 | -1 | 50 | 13 | 36 |
