# Supplementary material for: riboWaltz: Optimization of ribosome P-site positioning in ribosome profiling data
Source: PLoS Comput Biol. 2018 Aug 13;14(8):e1006169. doi: 10.1371/journal.pcbi.1006169 (PMC6112680; doi:10.1371/journal.pcbi.1006169)
Supplement: S5 Text — The PO computed from both read extremities are reported. The optimal PO used in the correction step of riboWaltz corresponds to 15 nucleotides from the 3’ end. (DOCX) [file pcbi.1006169.s018.docx]

| **Read length** | **riboWaltz** | | **RiboProfiling** | | **Plastid** | |
| --- | --- | --- | --- | --- | --- | --- |
|  | from 5’ end | from 3’ end | from 5’ end | from 3’ end | from 5’ end | from 3’ end |
| **20** | 11 | 8 | -23 | 42 | 13 | 6 |
| **21** | 8 | 12 | -10 | 30 | 13 | 7 |
| **22** | 11 | 10 | 19 | 2 | 13 | 8 |
| **23** | 7 | 15 | -29 | 51 | 13 | 9 |
| **24** | 8 | 15 | -10 | 33 | 13 | 10 |
| **25** | 9 | 15 | 18 | 6 | 13 | 11 |
| **26** | 10 | 15 | -17 | 42 | 13 | 12 |
| **27** | 11 | 15 | 2 | 24 | 38 | -12 |
| **28** | 12 | 15 | 3 | 24 | 9 | 18 |
| **29** | 13 | 15 | 13 | 15 | 10 | 18 |
| **30** | 13 | 16 | -8 | 37 | 24 | 5 |
| **31** | 15 | 15 | -22 | 52 | 13 | 17 |
| **32** | 16 | 15 | -27 | 58 | 13 | 18 |
| **33** | 14 | 18 | 11 | 21 | 13 | 19 |
| **34** | 18 | 15 | -19 | 52 | 13 | 20 |
| **35** | 16 | 18 | -47 | 81 | 13 | 21 |
| **37** | 12 | 24 | -34 | 70 | 13 | 23 |
| **38** | 20 | 17 | -24 | 61 | 13 | 24 |
| **40** | 22 | 17 | 20 | 19 | 13 | 26 |
| **41** | 15 | 25 | 27 | 13 | 13 | 27 |
| **42** | 23 | 18 | -1 | 42 | 13 | 28 |
| **43** | 23 | 19 | -31 | 73 | 13 | 29 |
| **44** | 21 | 22 | 6 | 37 | 13 | 30 |
| **46** | 30 | 15 | -15 | 60 | 13 | 32 |
