# Supplementary material for: riboWaltz: Optimization of ribosome P-site positioning in ribosome profiling data
Source: PLoS Comput Biol. 2018 Aug 13;14(8):e1006169. doi: 10.1371/journal.pcbi.1006169 (PMC6112680; doi:10.1371/journal.pcbi.1006169)
Supplement: S6 Text — The PO computed from both read extremities are reported. The optimal PO used in the correction step of riboWaltz corresponds to 13 nucleotides from the 5’ end. (DOCX) [file pcbi.1006169.s019.docx]

| **Read length** | **riboWaltz** | | **RiboProfiling** | | **Plastid** | |
| --- | --- | --- | --- | --- | --- | --- |
|  | from 5’ end | from 3’ end | from 5’ end | from 3’ end | from 5’ end | from 3’ end |
| **21** | 12 | 8 | 12 | 8 | 12 | 8 |
| **22** | 13 | 8 | 50 | 71 | 13 | 8 |
| **23** | 13 | 9 | 2 | 20 | 13 | 9 |
| **24** | 13 | 10 | 22 | 45 | 13 | 10 |
| **25** | 13 | 11 | 9 | 15 | 13 | 11 |
| **26** | 12 | 13 | 44 | 69 | 13 | 12 |
| **27** | 13 | 13 | 10 | 36 | 13 | 13 |
| **28** | 12 | 15 | 12 | 15 | 12 | 15 |
| **29** | 13 | 15 | 13 | 15 | 12 | 16 |
| **30** | 12 | 17 | 12 | 17 | 12 | 17 |
| **31** | 13 | 17 | 13 | 17 | 13 | 17 |
| **32** | 14 | 17 | 14 | 17 | 13 | 18 |
| **33** | 14 | 18 | 43 | 75 | 13 | 19 |
| **34** | 15 | 18 | 3 | 36 | 13 | 20 |
| **35** | 10 | 24 | 5 | 39 | 13 | 21 |
| **36** | 13 | 22 | 11 | 24 | 13 | 22 |
| **37** | 15 | 21 | 12 | 48 | 13 | 23 |
| **38** | 14 | 23 | 23 | 60 | 13 | 24 |
| **39** | 22 | 16 | 12 | 26 | 13 | 25 |
| **40** | 7 | 32 | 7 | 32 | 13 | 26 |
