# Supplementary material for: riboWaltz: Optimization of ribosome P-site positioning in ribosome profiling data
Source: PLoS Comput Biol. 2018 Aug 13;14(8):e1006169. doi: 10.1371/journal.pcbi.1006169 (PMC6112680; doi:10.1371/journal.pcbi.1006169)
Supplement: S7 Text — The PO computed from both read extremities are reported. The optimal PO used in the correction step corresponds to 12 nucleotides from the 5’ end. (DOCX) [file pcbi.1006169.s020.docx]

| Read  length | Number of reads (%) | Temporary P-site offset | | Corrected P-site offset | |
| --- | --- | --- | --- | --- | --- |
|  |  | from 5’ | from 3’ | from 5’ | from 3’ |
| **25** | 7.348 | 9 | 15 | 12 | 12 |
| **26** | 11.329 | 12 | 13 | 12 | 13 |
| **27** | 17.090 | 12 | 14 | 12 | 14 |
| **28** | 23.270 | 12 | 15 | 12 | 15 |
| **29** | 21.291 | 12 | 16 | 12 | 16 |
| **30** | 12.890 | 12 | 17 | 12 | 17 |
| **31** | 4.382 | 13 | 17 | 13 | 17 |
| **32** | 1.872 | 13 | 18 | 13 | 18 |
| **33** | 0.523 | 12 | 20 | 12 | 20 |
| **34** | 0.003 | 18 | 15 | 15 | 18 |
