# Supplementary material for: riboWaltz: Optimization of ribosome P-site positioning in ribosome profiling data
Source: PLoS Comput Biol. 2018 Aug 13;14(8):e1006169. doi: 10.1371/journal.pcbi.1006169 (PMC6112680; doi:10.1371/journal.pcbi.1006169)
Supplement: S8 Text — The PO computed from both read extremities are reported. The optimal PO used in the correction step corresponds to 11 nucleotides from the 5’ end. (DOCX) [file pcbi.1006169.s021.docx]

| Read  length | Number of reads (%) | Temporary P-site offset | | Corrected P-site offset | |
| --- | --- | --- | --- | --- | --- |
|  |  | from 5’ | from 3’ | from 5’ | from 3’ |
| **20** | 5.583 | 11 | 8 | 11 | 8 |
| **21** | 5.19 | 11 | 9 | 11 | 9 |
| **22** | 4.967 | 11 | 10 | 11 | 10 |
| **23** | 5.752 | 11 | 11 | 11 | 11 |
| **24** | 5.124 | 6 | 17 | 11 | 12 |
| **25** | 4.971 | 7 | 17 | 11 | 13 |
| **26** | 6.058 | 8 | 17 | 11 | 14 |
| **27** | 7.609 | 9 | 17 | 11 | 15 |
| **28** | 13.378 | 11 | 16 | 11 | 16 |
| **29** | 17.802 | 11 | 17 | 11 | 17 |
| **30** | 11.74 | 11 | 18 | 11 | 18 |
| **31** | 4.418 | 12 | 18 | 12 | 18 |
| **32** | 1.28 | 12 | 19 | 12 | 19 |
| **33** | 0.489 | 12 | 20 | 12 | 20 |
| **34** | 0.296 | 16 | 17 | 11 | 22 |
| **35** | 0.206 | 18 | 16 | 10 | 24 |
| **36** | 0.162 | 12 | 23 | 12 | 23 |
| **37** | 0.092 | 19 | 17 | 10 | 26 |
| **38** | 0.055 | 20 | 17 | 12 | 25 |
| **39** | 0.036 | 20 | 18 | 10 | 28 |
| **40** | 0.032 | 29 | 10 | 8 | 31 |
| **41** | 0.029 | 27 | 13 | 23 | 17 |
| **42** | 0.024 | 23 | 18 | 17 | 24 |
| **43** | 0.023 | 29 | 13 | 11 | 31 |
| **45** | 0.015 | 35 | 9 | 14 | 30 |
