# Supplementary material for: riboWaltz: Optimization of ribosome P-site positioning in ribosome profiling data
Source: PLoS Comput Biol. 2018 Aug 13;14(8):e1006169. doi: 10.1371/journal.pcbi.1006169 (PMC6112680; doi:10.1371/journal.pcbi.1006169)
Supplement: S9 Text — The PO computed from both read extremities are reported. The optimal PO used in the correction step corresponds to 11 nucleotides from the 5’ end. (DOCX) [file pcbi.1006169.s022.docx]

| Read  length | Number of reads (%) | Temporary P-site offset | | Corrected P-site offset | |
| --- | --- | --- | --- | --- | --- |
|  |  | from 5’ | from 3’ | from 5’ | from 3’ |
| **19** | 0.786 | 10 | 8 | 10 | 8 |
| **20** | 0.993 | 11 | 8 | 11 | 8 |
| **21** | 1.1 | 11 | 9 | 11 | 9 |
| **22** | 1.352 | 10 | 11 | 10 | 11 |
| **23** | 1.66 | 11 | 11 | 11 | 11 |
| **24** | 1.836 | 11 | 12 | 11 | 12 |
| **25** | 2.31 | 10 | 14 | 10 | 14 |
| **26** | 3.335 | 11 | 14 | 11 | 14 |
| **27** | 5.187 | 11 | 15 | 11 | 15 |
| **28** | 8.928 | 10 | 17 | 10 | 17 |
| **29** | 12.75 | 11 | 17 | 11 | 17 |
| **30** | 13.845 | 11 | 18 | 11 | 18 |
| **31** | 13.243 | 10 | 20 | 10 | 20 |
| **32** | 10.711 | 11 | 20 | 11 | 20 |
| **33** | 7.299 | 12 | 20 | 12 | 20 |
| **34** | 4.454 | 10 | 23 | 10 | 23 |
| **35** | 2.635 | 11 | 23 | 11 | 23 |
| **36** | 1.606 | 10 | 25 | 12 | 23 |
| **37** | 1.033 | 11 | 25 | 11 | 25 |
| **38** | 0.755 | 11 | 26 | 11 | 26 |
| **39** | 0.594 | 10 | 28 | 10 | 28 |
| **40** | 0.487 | 11 | 28 | 11 | 28 |
| **41** | 0.408 | 13 | 27 | 13 | 27 |
| **42** | 0.342 | 24 | 17 | 11 | 30 |
| **43** | 0.298 | 21 | 21 | 14 | 28 |
| **44** | 0.261 | 32 | 11 | 11 | 32 |
| **45** | 0.233 | 7 | 37 | 12 | 32 |
| **46** | 0.212 | 26 | 19 | 13 | 32 |
| **47** | 0.19 | 31 | 15 | 11 | 35 |
| **48** | 0.163 | 42 | 5 | 11 | 36 |
| **49** | 0.143 | 26 | 22 | 12 | 36 |
| **50** | 0.127 | 18 | 31 | 12 | 37 |
