# Supplementary material for: riboWaltz: Optimization of ribosome P-site positioning in ribosome profiling data
Source: PLoS Comput Biol. 2018 Aug 13;14(8):e1006169. doi: 10.1371/journal.pcbi.1006169 (PMC6112680; doi:10.1371/journal.pcbi.1006169)
Supplement: S10 Text — The PO computed from both read extremities are reported. The optimal PO used in the correction step corresponds to 11 nucleotides from the 5’ end. (DOCX) [file pcbi.1006169.s023.docx]

| Read  length | Number of reads (%) | Temporary P-site offset | | Corrected P-site offset | |
| --- | --- | --- | --- | --- | --- |
|  |  | from 5’ | from 3’ | from 5’ | from 3’ |
| **19** | 0.417 | 10 | 8 | 12 | 6 |
| **20** | 0.612 | 14 | 5 | 11 | 8 |
| **21** | 0.77 | 6 | 14 | 12 | 8 |
| **22** | 0.96 | 6 | 15 | 11 | 10 |
| **23** | 1.212 | 7 | 15 | 10 | 12 |
| **24** | 1.632 | 6 | 17 | 9 | 14 |
| **25** | 3.161 | 10 | 14 | 10 | 14 |
| **26** | 6.573 | 10 | 15 | 10 | 15 |
| **27** | 10.347 | 11 | 15 | 11 | 15 |
| **28** | 14.896 | 10 | 17 | 10 | 17 |
| **29** | 16.927 | 11 | 17 | 11 | 17 |
| **30** | 14.962 | 11 | 18 | 11 | 18 |
| **31** | 11.147 | 10 | 20 | 10 | 20 |
| **32** | 7.144 | 11 | 20 | 11 | 20 |
| **33** | 4.061 | 12 | 20 | 12 | 20 |
| **34** | 2.185 | 13 | 20 | 10 | 23 |
| **35** | 1.249 | 10 | 24 | 10 | 24 |
| **36** | 0.74 | 10 | 25 | 10 | 25 |
| **37** | 0.448 | 10 | 26 | 10 | 26 |
| **38** | 0.269 | 10 | 27 | 10 | 27 |
| **39** | 0.146 | 21 | 17 | 11 | 27 |
| **40** | 0.077 | 10 | 29 | 10 | 29 |
| **41** | 0.037 | 23 | 17 | 11 | 29 |
| **42** | 0.017 | 11 | 30 | 11 | 30 |
| **43** | 0.006 | 24 | 18 | 7 | 35 |
| **44** | 0.003 | 30 | 13 | 10 | 33 |
| **45** | 0.001 | 36 | 8 | 16 | 28 |
| **46** | 0 | 17 | 28 | 11 | 34 |
| **47** | 0 | NA | NA | 11 | 35 |
| **48** | 0 | NA | NA | 11 | 36 |
| **49** | 0 | 21 | 27 | 11 | 37 |
| **50** | 0 | NA | NA | 11 | 38 |
