# Supplementary material for: riboWaltz: Optimization of ribosome P-site positioning in ribosome profiling data
Source: PLoS Comput Biol. 2018 Aug 13;14(8):e1006169. doi: 10.1371/journal.pcbi.1006169 (PMC6112680; doi:10.1371/journal.pcbi.1006169)
Supplement: S11 Text — The optimal PO used in the correction step corresponds to 15 nucleotides from the 3’ end. (DOCX) [file pcbi.1006169.s024.docx]

| Read  length | Number of reads (%) | Temporary P-site offset | | Corrected P-site offset | |
| --- | --- | --- | --- | --- | --- |
|  |  | from 5’ | from 3’ | from 5’ | from 3’ |
| **20** | 0.874 | 13 | 6 | 11 | 8 |
| **21** | 0.932 | 6 | 14 | 8 | 12 |
| **22** | 1.127 | 6 | 15 | 11 | 10 |
| **23** | 1.365 | 7 | 15 | 7 | 15 |
| **24** | 1.551 | 8 | 15 | 8 | 15 |
| **25** | 2.527 | 9 | 15 | 9 | 15 |
| **26** | 8.375 | 10 | 15 | 10 | 15 |
| **27** | 30.555 | 11 | 15 | 11 | 15 |
| **28** | 32.89 | 12 | 15 | 12 | 15 |
| **29** | 15.461 | 13 | 15 | 13 | 15 |
| **30** | 2.878 | 13 | 16 | 13 | 16 |
| **31** | 0.433 | 15 | 15 | 15 | 15 |
| **32** | 0.143 | 11 | 20 | 16 | 15 |
| **33** | 0.08 | 17 | 15 | 14 | 18 |
| **34** | 0.033 | 18 | 15 | 18 | 15 |
| **35** | 0.019 | 16 | 18 | 16 | 18 |
| **37** | 0.008 | 26 | 10 | 12 | 24 |
| **38** | 0.01 | 30 | 7 | 20 | 17 |
| **40** | 0.017 | 17 | 22 | 22 | 17 |
| **41** | 0.022 | 15 | 25 | 15 | 25 |
| **42** | 0.023 | 30 | 11 | 23 | 18 |
| **43** | 0.02 | 25 | 17 | 23 | 19 |
| **44** | 0.019 | 25 | 18 | 21 | 22 |
| **46** | 0.003 | 25 | 20 | 30 | 15 |
