# Supplementary material for: riboWaltz: Optimization of ribosome P-site positioning in ribosome profiling data
Source: PLoS Comput Biol. 2018 Aug 13;14(8):e1006169. doi: 10.1371/journal.pcbi.1006169 (PMC6112680; doi:10.1371/journal.pcbi.1006169)
Supplement: S12 Text — The PO computed from both read extremities are reported. The optimal PO used in the correction step corresponds to 13 nucleotides from the 5’ end. (DOCX) [file pcbi.1006169.s025.docx]

| Read  length | Number of reads (%) | Temporary P-site offset | | Corrected P-site offset | |
| --- | --- | --- | --- | --- | --- |
|  |  | from 5’ | from 3’ | from 5’ | from 3’ |
| **21** | 11.8032 | 12 | 8 | 12 | 8 |
| **22** | 11.7396 | 13 | 8 | 13 | 8 |
| **23** | 5.318 | 13 | 9 | 13 | 9 |
| **24** | 2.2055 | 13 | 10 | 13 | 10 |
| **25** | 1.6783 | 11 | 13 | 13 | 11 |
| **26** | 1.528 | 12 | 13 | 12 | 13 |
| **27** | 3.2145 | 11 | 15 | 13 | 13 |
| **28** | 11.8113 | 12 | 15 | 12 | 15 |
| **29** | 16.3201 | 13 | 15 | 13 | 15 |
| **30** | 13.6047 | 12 | 17 | 12 | 17 |
| **31** | 7.9517 | 13 | 17 | 13 | 17 |
| **32** | 2.9854 | 27 | 4 | 14 | 17 |
| **33** | 1.5143 | 27 | 5 | 14 | 18 |
| **34** | 0.0263 | 12 | 21 | 15 | 18 |
| **35** | 0.0258 | 3 | 31 | 10 | 24 |
| **36** | 0.0184 | 11 | 24 | 13 | 22 |
| **37** | 0.0133 | 29 | 7 | 15 | 21 |
| **38** | 0.0101 | 30 | 7 | 14 | 23 |
| **39** | 0.0069 | 22 | 16 | 22 | 16 |
| **40** | 0.0052 | 30 | 9 | 7 | 32 |
