# Supplementary material for: riboWaltz: Optimization of ribosome P-site positioning in ribosome profiling data
Source: PLoS Comput Biol. 2018 Aug 13;14(8):e1006169. doi: 10.1371/journal.pcbi.1006169 (PMC6112680; doi:10.1371/journal.pcbi.1006169)
Supplement: S13 Text — (DOCX) [file pcbi.1006169.s026.docx]

*RiboSeq data processing*

Raw reads were processed by removing 5’ adapters, discarding reads shorter than 20 nucleotides and trimming the first nucleotide (using Trimmomatic v0.36). Reads mapping on rRNAs and tRNAs (downloaded from the SILVA rRNA and the Genomic tRNA databases respectively) were removed. The remaining reads were aligned to the organism transcriptome with Bowtie2 (v2.2.6) employing the default settings. All reads aligning to the very same region were collapsed to avoid potential PCR duplicates, and only strand-specific reads were kept.
